# Protective Effects of a Polyherbal Mixture on Intestinal Injury via the NF-κB Signaling Pathway and Gut Microbiota Modulation in Hyperuricemic Mice

**DOI:** 10.3390/foods14071118

**Published:** 2025-03-24

**Authors:** Haoluan Wang, Yu Xi, Fengju Gu, Linlin Peng, Jian Li

**Affiliations:** 1 Key Laboratory of Geriatric Nutrition and Health, Ministry of Education, Beijing Technology and Business University, Beijing 100048, China; 15511316985@163.com (H.W.); gu17861820903@163.com (F.G.); 19834545170@163.com (L.P.); 2 Key Laboratory of Green and Low-Carbon Processing Technology for Plant-Based Food of China National Light Industry Council, School of Food and Health, Beijing Technology and Business University, Beijing 100048, China

**Keywords:** polyherbal mixture, hyperuricemia, gut microbiota, intestinal barrier, NF-κB signaling pathway

## Abstract

This study investigated the protective effects of a polyherbal tea (PHT) on intestinal injury in hyperuricemia (HUA) mice and the underlying mechanisms. PHT was orally administered to mice for 49 days, while potassium oxonate and hypoxanthine were administered 7 days after PHT administration and continued for 42 days to cause HUA. Treatment with PHT significantly reduced serum uric acid and blood urea nitrogen levels in HUA mice. It also inhibited liver xanthine oxidase activity and promoted intestinal uric acid excretion through the upregulation of transporters GLUT9 and ABCG2. Intestinal barrier integrity was reinforced, as evidenced by the restoration of the villous structure, reduction in edema, and upregulation of tight junction proteins (occludin, ZO-1) and mucin (MUC2). Moreover, PHT suppressed serum LPS levels and inhibited the NF-κB pathway, leading to a reduction in TNF-α and IL-6 levels in the gut. Gut microbiota analysis revealed PHT reversed dysbiosis, enriching beneficial bacteria like *Duncaniella* sp. and *Heminiphilus faecis*. By UPLC–MS analysis, 154 compounds of PHT persisted in the gut, suggesting that these compounds are likely to modulate both intestinal barrier function and gut microbiota. These findings suggest that this PHT may have potential as a functional food for the prevention of hyperuricemia.

## 1. Introduction

Hyperuricemia (HUA) is a disorder of purine metabolism in the body that leads to excessive production or impaired excretion of uric acid, resulting in abnormally high levels of uric acid in the blood [1]. Recently, the global prevalence of HUA has increased and is trending toward younger age groups [2]. Epidemiologic surveys have shown that the prevalence of HUA ranges from 10.5% to 16.6% in Australia [3], approximately 14% in China [4], and as high as 20.7% in the United States [5], making HUA the second most important metabolic disease threatening human health after diabetes. Studies have shown that HUA is often accompanied by pathological processes such as kidney disease, cardiovascular disease, diabetes, and metabolic syndrome [6,7,8]. When blood levels of uric acid are too high, the deposition of uric acid crystals in the joints can lead to gout. In addition, HUA affects gut health, leading to dysbacteriosis and the production of inflammation-related metabolites; simultaneously, HUA may impair the intestinal excretion of uric acid, thereby aggravating intestinal microecological disturbances [9].

The intestine plays a crucial role in uric acid homeostasis, accounting for approximately one-third of uric acid excretion [10]. Uric acid transport across the intestinal epithelium is regulated by key transporters, such as GLUT9 and ABCG2 [11,12]. However, efficient uric acid excretion requires an intact intestinal barrier. Damage to intestinal villi, reduced expression of tight junction proteins (occludin and ZO-1), and decreased mucin (MUC2) production lead to increased intestinal permeability and a destabilized gut microenvironment [13,14]. Chronic inflammation further exacerbates intestinal barrier dysfunction, disrupting epithelial integrity and enhancing gut permeability, which, in turn, contributes to the progression of hyperuricemia [15]. The nuclear factor-kappa B (NF-κB) signaling pathway is a key regulator of intestinal inflammation and barrier integrity [16]. Once activated by bacterial toxins and proinflammatory stimuli, the inhibitory protein IκBα in the cytoplasm is phosphorylated and degraded, and the NF-κB p65 subunit is then translocated to the nucleus, leading to the transcription of proinflammatory cytokines [17]. Targeting NF-κB signaling has therefore been proposed as a promising approach to mitigate intestinal injury associated with HUA.

The gut microbiota also plays a pivotal role in HUA pathophysiology [18,19]. Certain probiotic strains have been shown to modulate uric acid metabolism. For instance, *Lactobacillus plantarum strains* LLY-606 and X7022 effectively improve gut dysbiosis [20,21], while *Lacticaseibacillus paracasei* MJM60396 reduces uric acid synthesis by absorbing purines and inhibiting xanthine oxidase (XOD) activity, as well as enhancing intestinal barrier integrity [22]. Additionally, *Lacticaseibacillus rhamnosus* 1155 inhibits hepatic and serum XOD activity while upregulating ABCG2 expression in intestinal tissues, promoting uric acid excretion and restoring microbial balance [23]. Certain lactic acid bacteria, *Bifidobacterium* species, and other microbes contribute to intestinal epithelial energy supply through the production of butyric acid and an enhancement in excretory processes [24]. Furthermore, *Limosilactobacillus fermentum* 2644 enhances intestinal barrier integrity by upregulating occludin and MUC2 expression while suppressing lipopolysaccharide (LPS) induced inflammatory responses and reducing interleukin-1β (IL-1β) levels [23]. Severe dysbiosis in hyperuricemic conditions aggravates inflammation, further suppressing uric acid transporters and establishing a detrimental feedback loop that worsens disease progression [25]. Given these, maintaining intestinal barrier integrity and regulating gut microbiota composition have emerged as potential therapeutic strategies for alleviating HUA.

Recent studies have demonstrated that food and medicine homology and polyherbal mixtures hold significant potential in the management of hyperuricemia (HUA) due to their mild nature and multi-target synergistic effects [26]. Polyherbal mixtures, such as *Ganoderma lucidum* cultures and the traditional Chinese remedy “ShiZhiFang”, have exhibited notable uric acid-lowering effects in both preclinical and clinical settings [27,28]. Similarly, Cheqianzi Decoction (CQD), a polyherbal mixture, has been shown to activate renal ABCG2 transporters, thereby facilitating uric acid excretion and modulating inflammatory signaling pathways [29]. Euodiae fructus (Wuzhuyu) has been reported to suppress renal inflammation as well as reduce uric acid production and enhance its excretion [30]. While these mixtures have demonstrated efficacy in lowering uric acid levels and alleviating renal dysfunction, their role in protecting against HUA-induced intestinal injury remains largely unexplored. Given the crucial role of the intestine in uric acid metabolism, investigating the potential of polyherbal formulation interventions to preserve intestinal integrity is essential for a more comprehensive hyperuricemia management strategy.

To address this gap, the present study focused on the potential intestinal protective effects of a polyherbal tea (PHT), a food and medicine homology mixture composed of green tea, *Smilax glabra* (Smilacis glabrae rhizome), *Dioscorea opposita* (Dioscoreae rhizome), *Glehnia littoralis* (Glehniae radix), *Senna tora* (Cassiae semen) and *Senna alexandrina* (Sennae folium). Most of these ingredients have been individually studied for their beneficial effects on hyperuricemia. For instance, polyphenols like epigallocatechin gallate (EGCG) in green tea inhibit XOD activity, thereby reducing uric acid production [31,32]. Flavonoids from *Smilax glabra* have been shown to suppress XOD activity and modulate inflammation [33,34], while saponins from *Dioscorea opposita* enhance uric acid excretion by regulating transporters such as GLUT9 and ABCG2 [35]. Moreover, anthraquinones from *Senna tora* not only promote uric acid excretion but also suppress NF-κB signaling, which may help alleviate HUA-induced intestinal injury [36,37].

Unlike probiotics, which influence intestine health primarily through microbial interactions, PHT provides bioactive compounds that may exert direct effects on intestinal barrier integrity, uric acid transport, and inflammation. While probiotics depend on colonization and the host microbiome composition, PHT offers an alternative approach that may complement microbial interventions in hyperuricemia management. Given that these bioactive compounds must remain stable within the gut to exert their effects, understanding their persistence and metabolism is critical for evaluating their therapeutic potential. Previous studies have demonstrated that metabolomics approaches, particularly ultra-performance liquid chromatography–mass spectrometry (UPLC-MS), provide a powerful tool for detecting, characterizing, and quantifying bioactive compounds in complex biological matrices, including the gut environment [38,39]. By employing UPLC-MS metabolomics, this study aims to assess the persistence of bioactive compounds within the gut. This approach will help clarify the mechanisms by which these compounds exert their biological effects and contribute to host health.

Therefore, the present study was conducted to establish an HUA model in mice induced with potassium oxonate (PO) in combination with hypoxanthine (HX) and to evaluate the role and molecular mechanism of PHT in the prevention of intestinal injury in HUA mice. We systematically investigated the regulatory effects of PHT on systemic uric acid metabolism, intestinal barrier function, intestinal inflammation, and intestinal microecology using multiple approaches. Furthermore, we preliminarily identified the bioactive compounds retained in the small intestine following PHT administration using UPLC-MS. This study provides an important theoretical and applied basis for the further development of medicinal foods with the potential to lower uric acid levels and protect intestinal function.

## 2. Materials and Methods

### 2.1. Chemicals and Materials

Potassium oxonate (PO, purity > 98%), hypoxanthine (HX, purity > 99%), and sodium carboxymethyl cellulose (CMC-Na) were purchased from Shanghai Yuanye Biotechnology Co., Ltd. (Shanghai, China). Assay kits for uric acid were obtained from the Jiancheng Bioengineering Institute (Nanjing, China). Blood urea nitrogen (BUN) and xanthine oxidase (XOD) test kits were provided by Beijing Suo Laibao Technology Co., Ltd. (Beijing, China). LPS content was determined using an ELISA kit purchased from Kotex Bio Co., Ltd. (Suzhou, China).

### 2.2. Preparation of PHT

PHT (Besunyen Detox Tea) was purchased from Beijing Aote Shuer Health Products Development Co., Ltd. (Beijing, China). It mainly contains green tea, *Smilax glabra* (Smilacis glabrae rhizome), *Dioscorea opposita* (Dioscoreae rhizome), *Glehnia littoralis* (Glehniae radix), *Senna tora* (Cassiae semen), and *Senna alexandrina* (Sennae folium).

### 2.3. Animals and Experimental Design

Thirty male ICR mice (26 ± 2 g, 8 weeks old) were provided by SPF Biotechnology Co., Ltd. (Beijing, China), animal License No. SYXK (Beijing) 2022-0021. The mice were bred at Zhongyan Zichuang Biotechnology Co., Ltd. (Beijing, China), and the experimental protocol was approved by the Scientific Research Ethics Committee of Beijing Technology and Business University (15 April 2024 No. 123). Before the start of the experiment, all the mice were housed in a room with a humidity of 60% and a temperature of 22–25 °C on a 12 h light–dark cycle and were given free access to food and water throughout this study. After 1 week of environmental adjustment, the mice were randomly divided into 5 groups (*n* = 6/group): the control group (Control), HUA model group (HUA), PHT low-dose group (HUA+L), PHT medium-dose group (HUA+M), and PHT high-dose group (HUA+H).

**Figure 1:**
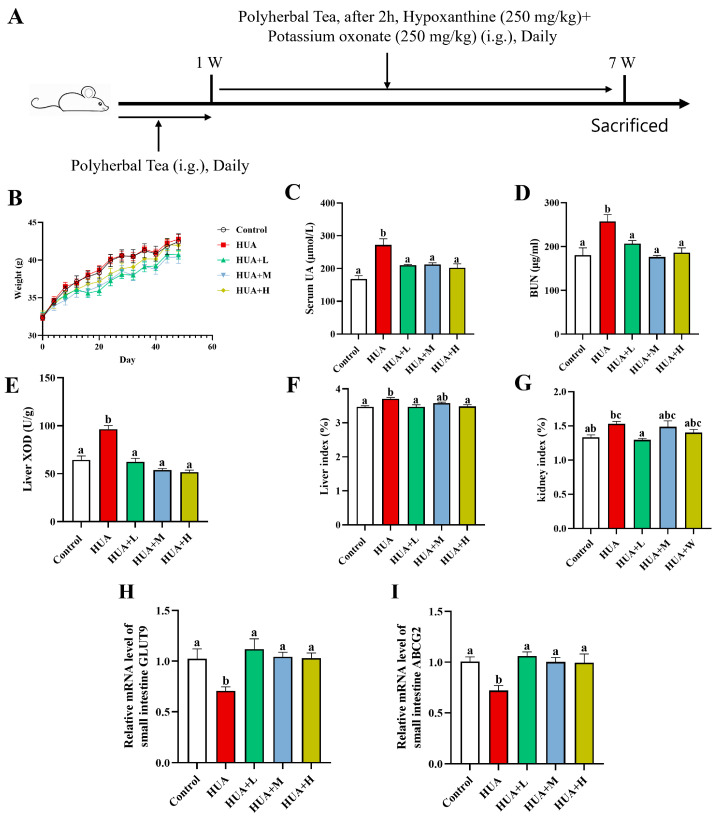
Intervention effect of PHT on hyperuricemic mice. (**A**) Schematic illustration of the experimental procedure: the mice were given hypoxanthine (250 mg/kg) and potassium oxonate (250 mg/kg) to induce hyperuricemia by continuous gavage of PHT for 1 week until the end of the 7th week when the mice were sacrificed. (**B**) Mouse body weight change curve. (**C**) Serum uric acid (UA) level. (**D**) Serum urea nitrogen (BUN) level. (**E**) Liver xanthine oxidase (XOD) activity. (**F**) Liver index. (**G**) Kidney index. RT-qPCR was used to detect the expression of uric acid transporter protein mRNA in the small intestine of the mice in each group. Small intestine (**H**) GLUT9, (**I**) ABCG2. Data are expressed as mean ± SEM (*n* = 6). Different letters (a–c) indicate significant differences between groups of the same index (*p* < 0.05).

The intervention plan for animal experiments is shown in Figure 1A. Both the Control and Model groups were provided with ultrapure water, while the PHT-treated groups were administered different doses of PHT extract via gavage. The maximum recommended human intake of PHT is the infusion prepared from 5 g of PHT in boiling water per day (https://ts.cqszzs.com/baike/bjsp/379.html (accessed on 1 November 2023)). Using the Xu method, the corresponding gavage dose for mice was calculated to be 0.65 mg/g BW/day [40]. Thus, the mice in the PHT-L, PHT-M, and PHT-H groups were administered with 0.325, 0.65, and 1.30 mg PHT/g BW/day, respectively. To ensure accurate and consistent administration, PHT was infused in boiling water, filtered, and prepared as an aqueous extract. The extract was administered via gavage at a standardized volume of 0.1 mL per 10 g BW, with the final prepared extract concentrations corresponding to 0.0325 g/mL (PHT-L), 0.065 g/mL (PHT-M), and 0.130 g/mL (PHT-H). The prepared solutions were aliquoted into single-use tubes and stored at −20 °C, protected from light. Prior to administration, aliquots were thawed at 4 °C for 12 h, vortexed, and equilibrated to 25 °C. The mice received daily oral gavage of freshly thawed solutions using sterile feeding needles. No aliquot underwent repeated freeze–thaw cycles.

The HUA model was established as previously reported, with some modifications [41,42]. PO and HX were dissolved in 0.5% sodium carboxymethyl cellulose (CMC-Na). To fully demonstrate the potential of PHT in the early prevention of HUA, the experiment was initiated by gavage 7 d prior to the induction of modeling so that the body could first adapt to the active ingredients in the tea and establish homeostasis. For the following 42 consecutive days, the mice in the control and HUA groups received daily oral gavage of distilled water, while the tea-treated groups were administered the same volume of tea water extract at the corresponding concentration via gastric gavage needle. After 2 h, the HUA, low, medium, and high groups received combined treatment with PO (250 mg/kg) and HX (250 mg/kg), whereas the control group received 0.5% CMC-Na. At the end of the experiment, materials were taken from the anesthetized mice, including eye removal for blood, collection of the intestinal contents, removal of the intestinal tissue, and collection of fecal samples. The blood samples were then centrifuged at 4000 r/min at 4 °C for 10 min to obtain the serum. The organ index was calculated by weighing the collected organs before they were stored at −80 °C. An organ index was calculated based on the following formulas [43]:

(1) $$\mathrm{Liver}\;\mathrm{index}=\frac{liver\;weight}{Body\;weight}\times 100\%$$

(2) $$\mathrm{kidney}\;\mathrm{index}=\frac{kidney\;weight}{Body\;weight}\times 100\%$$

### 2.4. Histological Analysis

Jejunum, ileum, and colon tissues were collected and fixed in 10% formalin buffer. The samples were then embedded in paraffin after dehydration. The tissues were subsequently cut into 5 μm thick slices and stained with hematoxylin and eosin (H&E). The samples were scanned with a panoramic scanner for observation. Histologic scoring of the jejunum and ileum was performed using Chiu’s scale [44,45]. The degree of colonic injury was scored according to previous studies [46].

### 2.5. Real-Time Quantitative PCR (RT–qPCR) Analysis

Total RNA was extracted from the small intestine according to the instructions of the TransZol Up Plus RNA Kit (TransGen Biotech, Beijing, China). The RNA samples were subsequently reverse-transcribed into cDNA using a HiScript IV All-in-One Ultra RT SuperMix for qPCR kit (Novozymes Biotechnology Co., Ltd., Nanjing, China). RT–qPCR was performed on an ABI Quant Studio 3 instrument using AceQ qPCR SYBR Green Master Mix (Novozymes Biotechnology Co., Ltd., Nanjing, China). Relative gene expression levels were determined using the 2^−ΔΔCt^ method. The β-actin gene was used as an internal control. The sequences of the target genes and reference primers are shown in Table 1.

### 2.6. Western Blotting

Small intestine tissue proteins were extracted using RIPA lysis buffer purchased from Servicebio (Wuhan, China) with a Roche protease inhibitor and phosphatase inhibitor. The protein concentrations of the lysates were quantified using a BCA protein assay kit (Beyotime Biotechnology Co., Ltd., Shanghai, China). The extracted proteins were isolated with 12.5% sodium dodecyl sulfate–polyacrylamide gel electrophoresis (SDS-PAGE) and transferred to a PVDF membrane. The PVDF membrane was blocked with 5% skim milk solution for 2 h. The membrane was rinsed with TBST buffer to remove skim milk residues and incubated overnight at 4 °C with the following primary antibodies: anti-NFκB-p65 (1:1000 dilution; #8242; Cell Signaling Technology, Inc., Danvers, MA, USA), anti-p-NFκB-p65 (1:1000 dilution; AP0999; ABclonal Technology Co., Ltd., Wuhan, Hubei, China), anti-TNF-α (1:1000 dilution; A20851; ABclonal Technology), anti-p-IκBα (1:1000 dilution; #9246; Cell Signaling Technology), anti-IκBα (1:7000 dilution; A24909; ABclonal Technology), and anti-β-actin (1:5000 dilution; 20536-1-AP; Proteintech Group, Inc., Rosemont, IL, USA). The following day, the samples were incubated again for 1 h at room temperature with the corresponding secondary antibody. The membranes were covered with ECL chemical solution, imaged using an automated chemiluminescence imaging system (Tanon 5200, Shanghai Tianneng Technology Co., Ltd., Shanghai, China), and analyzed for optical density using ImageJ (version 6.0.)

### 2.7. Gut Microbial Analysis

After the mice were dissected, their feces were collected aseptically and immediately frozen in liquid nitrogen for subsequent gut microbiota analysis. The following sequencing steps were performed by Shanghai Zhongke New Life Biotechnology Co., Ltd. (Shanghai, China). After completing genomic DNA extraction, the extracted genomic DNA was detected using 1% agarose gel electrophoresis. The fragments were approximately 350 bp. PE libraries were constructed as follows: (1) “Y” joints were connected, (2) junction self-associated fragments were removed via magnetic bead screening, (3) library templates were enriched via PCR amplification, and (4) sodium hydroxide was used to denature DNA and produce single-stranded DNA fragments. For bridge PCR, (1) one end of the DNA fragment complementary to the primer base was immobilized on the chip; (2) the other end was randomly complementary to another nearby primer, which was also immobilized, forming a “bridge”; (3) PCR amplification was performed to produce DNA clusters; and (4) the DNA amplicon was linearized into a single strand. For Illumina sequencing, (1) modified DNA polymerase and dNTPs with 4 fluorescent markers were added to synthesize one base per cycle. (2) The surface of the reaction plate was scanned with a laser to read the type of nucleotide that was polymerized by the first round of reactions for each template sequence. (3) Chemical cleavage of the “fluorescent group” and the “termination group” restored the stickiness of the 3′ end, and the polymerization of the second nucleotide continued. (4) The fluorescent signals collected in each round were counted to obtain the sequence of the template DNA fragment. The original sequences were optimized for splitting, mass shearing, and contamination removal. The optimized sequences were then used for splice assembly and gene prediction, and the resulting genes were annotated to species and classified. ORF prediction of contigs in the splicing results was performed using MetaGene (https://www.metagene.de/ (accessed on 10 August 2024)). The gene sequences predicted from all the samples were clustered (default parameters: 90% identity and 90% coverage) using CD-HIT software (version 4.8.1), and the longest gene in each class was taken as the representative sequence to construct a nonredundant gene set. High-quality reads from each sample were individually compared with the nonredundant gene set using SOAPaligner software (version 2.21) to characterize the abundance of genes in the corresponding samples statistically. The predicted genes were classified by species annotation and analyzed by statistical comparison of sample similarity clustering differences.

### 2.8. UPLC–MS Analysis of PHT and Cecum Contents

For pretreatment with PHT, 400 μL of tea water extract was vacuum-dried. Two hundred microliters of 40% methanol aqueous solution were added for redissolution. The mixture was vortexed for 30 s, solubilized via ultrasonication, and centrifuged for 20 min (16,000× *g*, 4 °C), after which the supernatant was collected. To pretreat the cecum contents, the samples were weighed, precooled with 70% methanol, sonicated for 20 min in a cold-water bath, and centrifuged at 16,000× *g* and 4 °C for 20 min, after which the supernatants were collected. After vacuum drying, 100 μL of 40% aqueous methanol solution was added to the residue, which was subsequently vortexed and centrifuged at 16,000× *g* and 4 °C for 15 min, after which the supernatant was collected.

An Orbitrap Exploris™ 480 mass spectrometer (Thermo Fisher Scientific, Bremen, Germany) coupled with an ultrahigh-performance liquid chromatography (UHPLC) system was used for the identification of PHT and cecum contents.

The chromatographic conditions used an ACQUITY UPLC HSS T3 column (2.1 mm × 100 mm, 1.8 µm) at 35 °C with a sample size of 2 μL. Mobile phase A (containing 0.1% formic acid solution) and mobile phase B (containing 0.1% formic acid acetonitrile solution) were used at a flow rate of 0.3 mL/min. The elution gradient was as follows: isocratic elution with 5% B was performed for 1 min, and then the percentage of B was increased to 98% in 16 min. Next, the proportion of B was adjusted back to 5% in 0.5 min, and finally, another 2.5 min of isocratic elution at 5% B was performed.

Mass spectrometry analysis was performed using electrospray ionization (ESI) in positive and negative ion modes. The ESI source parameters were as follows: spray voltage, 3800 V (ESI+)/3500 V (ESI-); sheath gas flow, 45 L/min; ion transfer tube temperature, 320 °C; and atomization temperature, 350 °C. Detection was performed in full scan/data-dependent secondary scan mode with the top 10 MS1 ions to obtain MS/MS spectra. The collision energies (CEs) were in step-normalized energy levels of 20, 40, and 60; the primary mass–charge ratio scanning range was 90–1300.

### 2.9. Statistical Analysis

The data are expressed as the mean ± standard error of the mean (SEM). A one-way ANOVA (and Tukey’s multiple comparison test for all groups) was performed using GraphPad Prism v6.01 and SPSS software v23.0. Differences were considered statistically significant when *p* < 0.05.

## 3. Results

### 3.1. PHT Alleviated HUA Induced by Potassium Oxonate and Hypoxanthine

Given that the effects of PHT may require time to accumulate and modulate conditions, the design of a tea intervention for a sustained period of 1 week prior to PO+HX induction may more realistically imitate the scenario of early intake of functional foods for the prevention of HUA in the population. As shown in Figure 1A, HUA was induced by combining HX and PO after continuous gavage of PHT to mice for 1 week until the end of the 7th week when the material was collected. Body weight monitoring (Figure 1B) showed that the body weights of the mice in all the groups increased steadily over time, and there was no significant difference between the groups (*p* > 0.05), suggesting that the different doses of PHT did not negatively affect the growth status of the mice. The serum uric acid (UA) level was significantly higher in the HUA group than in the control group (an increase of 61.9%; Figure 1C), indicating the successful establishment of the HUA model. Compared with that of the HUA-treated mice, the serum UA levels of the HUA-treated mice decreased by 22.9%, 21.7%, and 25.6% (*p* < 0.05) after PHT intervention at low (0.325 mg PHT/g BW/day), medium (0.650 mg PHT/g BW/day), or high (1.300 mg PHT/g BW/day) doses, respectively. Additionally, serum urea nitrogen (BUN) was significantly higher in the HUA group than in the control group (Figure 1D). All three doses of PHT significantly reduced BUN levels and achieved 19.5%, 31.5%, and 27.6% reductions, respectively, essentially returning them to normal levels. The PHT group was still able to significantly reduce serum UA after induction, suggesting that early intervention blocked the development of HUA to some extent, which is consistent with the original intent of the preventive design. The results of the liver XOD activity assay (Figure 1E) showed that the HUA group presented a 49.5% increase in XOD activity compared with that of the control group, whereas low, medium, and high doses of PHT reduced XOD activity by 35.4%, 44.1% and 46.5%, respectively, and restored XOD activity to a level similar to that of the control group (*p* < 0.05), suggesting that PHT can alleviate HUA by inhibiting the key enzymes involved in uric acid synthesis. Compared with those in the control group, both the liver and kidney indices (Figure 1F,G) were also elevated to some extent in the HUA group; the difference in the liver indices was significant, whereas the difference in the kidney indices was not. All three doses of PHT tended to decrease the liver indices but had a weaker effect on the kidney indices. To further explore the potential role of PHT in the route of intestinal excretion, we examined the mRNA levels of the uric acid transporter proteins GLUT9 and ABCG2 in the small intestine (Figure 1H,I). The results showed that GLUT9 was downregulated by 34.3% in the HUA group compared with that of the control group, whereas it was upregulated by 76.5% after the low-dose tea treatment and recovered to the control level. ABCG2 expression also significantly reversed from a 21.9% decrease in the HUA group to a 25.1% increase with PHT (*p* < 0.05). It is evident that PHT also promotes improvement in the uric acid excretion pathway by upregulating the intestinal uric acid transporter genes.

In conclusion, PHT significantly inhibited UA elevation, BUN accumulation, and excessive enhancement in XOD activity in the hyperuricemic mice at all three doses and also promoted uric acid excretion by upregulating the expression of intestinal uric acid transporter proteins (GLUT9 and ABCG2), suggesting that PHT significantly prevents and improves HUA. Because there was no statistically significant difference among the low-, medium-, and high-dose groups in terms of improvement in the above indices, based on economy and safety, a low dose (HUA+L, 0.325 mg PHT/g BW/day) was chosen for further investigation.

### 3.2. PHT Improved Intestinal Injury and Maintained Barrier Integrity

To evaluate the protective effects of PHT on intestinal tissue structure and barrier function under HUA, we performed H&E staining; recorded the histological injury scores of the ileum, jejunum, and colon of mice; and measured the expression levels of key genes related to the intestinal barrier (occludin, ZO-1, and MUC2).

**Figure 2:**
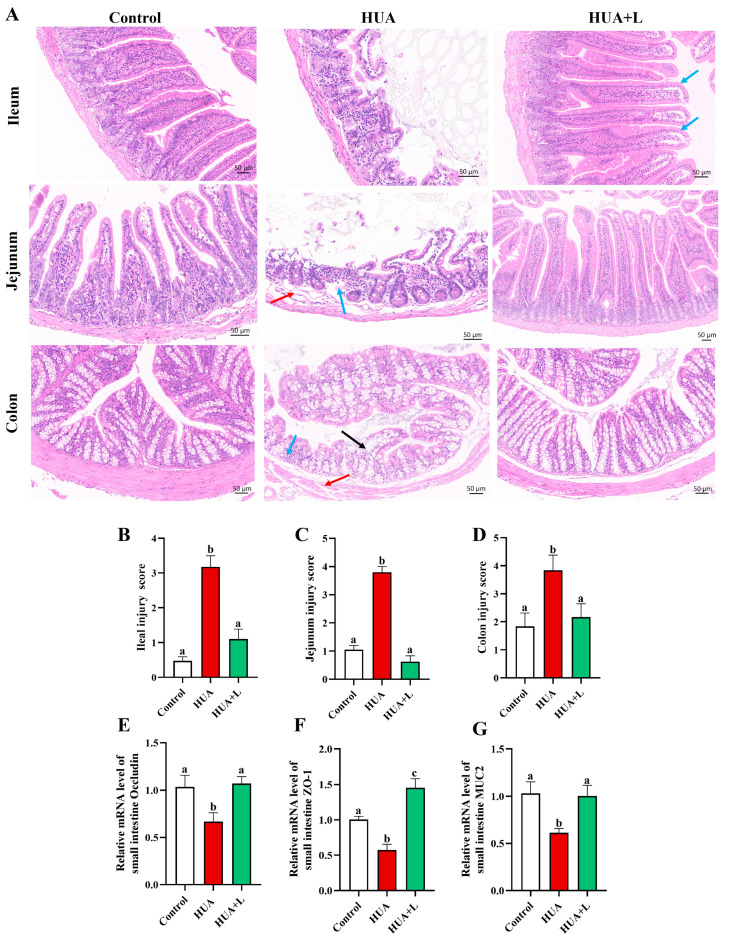
PHT maintains intestinal barrier integrity in hyperuricemic mice. (**A**) Representative diagrams of H&E staining of the jejunum, ileum, and colon in each group of mice and histologic scores (**B**–**D**). RT-qPCR was used to detect the expression of mRNA of small intestinal barrier-related genes in each group of mice. (**E**) Occludin. (**F**) ZO-1. (**G**) MUC2. Data are expressed as mean ± SEM (*n* = 6). Different letters (a–c) indicate significant differences between groups for the same indicator (*p* < 0.05).

As shown in Figure 2A, the ileum and jejunum of the control group showed a complete and neat villous structure with shallow and well-defined crypts. The epithelial and mucosal layers of the colon were well arranged. In contrast, the HUA group showed a large apical absence of intestinal villi, a significant increase in crypt depth, intrinsic membrane disintegration (blue arrows) and submucosal edema (red arrows) in the jejunum, and a disorganized epithelial structure of the colon (black arrows) with lymphocyte aggregation (blue arrows). The colonic submucosa also exhibits edema (red arrows). These findings suggest that HUA causes significant injury to the mouse intestine. After the low-dose PHT (HUA+L) intervention, the integrity of the villus structures of the ileum and jejunum was largely restored, and the depth of the crypts was significantly reduced. The mucosal epithelium of the colon was also partially repaired, and only mild edema was observed in the submucosa. The histologic scoring results validated this trend: compared with those of the control group, the respective ileum and jejunum injury scores increased to 3.17 and 3.8 in the HUA group, whereas the PHT treatment resulted in significant decreases of 65.3% and 83.4% (*p* < 0.05). In the colon, the degree of injury was 52.2% greater in the HUA group than in the control group and decreased by 43.3% after the tea intervention. These results indicate that PHT has favorable reparative or preventive effects on both the small intestine and colon of mice.

To further explore the barrier protection effect at the molecular level, we examined the mRNA levels of the occludin, ZO-1, and MUC2 genes, which are associated with tight junctions and mucus secretion in the small intestine (Figure 2E–G). The results showed that the expressions of these three genes in the HUA group were significantly lower than those in the control group (*p* < 0.05), whereas the PHT intervention upregulated them by 60.6%, 153.2%, and 63.6%, respectively, to reach or exceed normal levels. PHT ameliorated intestinal mucosal injury not only morphologically but also significantly maintained intestinal barrier integrity in the HUA mice by increasing the expression of tight junction proteins and mucins.

### 3.3. PHT Relieved Intestinal Inflammation in Hyperuricemic Mice

**Figure 3:**
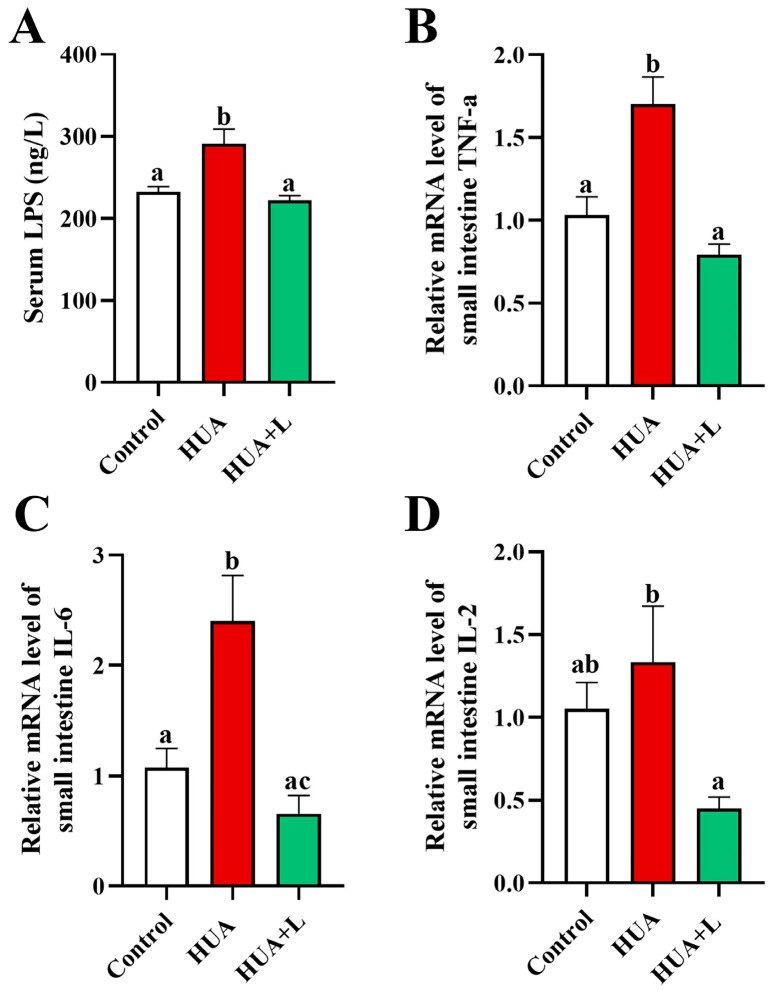
Effect of PHT on intestinal inflammation in hyperuricemic mice. (**A**) Serum LPS levels in mice. RT-qPCR was used to detect the expression of small intestinal inflammatory factors (**B**) TNF-α, (**C**) IL-6, and (**D**) IL-2 mRNA in each group of mice. Data are expressed as mean ± SEM (*n* = 6). Different letters (a–c) indicate significant differences between groups for the same indicator (*p* < 0.05).

In the intestinal environment, impaired barrier function is often mutually reinforcing with inflammatory responses. Decreased expression of epithelial tight junction proteins (occludin and ZO-1) and mucus layer proteins (MUC2) increases permeability, allowing leakage of microbial toxins (e.g., LPS) and aggravating mucosal inflammation. Persistent intestinal inflammation can also destroy the barrier structure, creating a positive feedback cycle [47,48,49]. To further evaluate the anti-inflammatory and barrier-protective effects of PHT in hyperuricemic mice, we focused on serum LPS levels and the expression of major proinflammatory cytokines. As shown in Figure 3A, serum LPS levels in the HUA group increased from 232.1 to 291.1 ng/L (a 25.4% increase). After the low-dose PHT (HUA+L) intervention, the LPS levels were reduced by 69.4 ng/L and did not differ significantly (*p* > 0.05) from those of the control group. These findings suggest that tea interventions may, to some extent, inhibit the possible increase in LPS leakage or accumulation due to barrier impairment. Moreover, TNF-α and IL-6 mRNA expression in the small intestine tissues of the HUA group increased by 1.65- and 2.23-fold, respectively, compared with those of the control group (Figure 3B,C). After PHT intervention, both TNF-α and IL-6 mRNA levels significantly decreased and recovered to the levels of the control group (*p* > 0.05). In addition, IL-2 mRNA expression in the HUA group was slightly higher than that in the control group but not significantly different and was further downregulated after the tea intervention (Figure 3D). In conclusion, PHT can downregulate the expression of various proinflammatory factors in the gut of HUA mice and reduce the level of LPS, thus alleviating the localized inflammatory response caused by dysfunction of the intestinal barrier.

### 3.4. PHT Inhibited the NF-κB Signaling Pathway in the Gut

**Figure 4:**
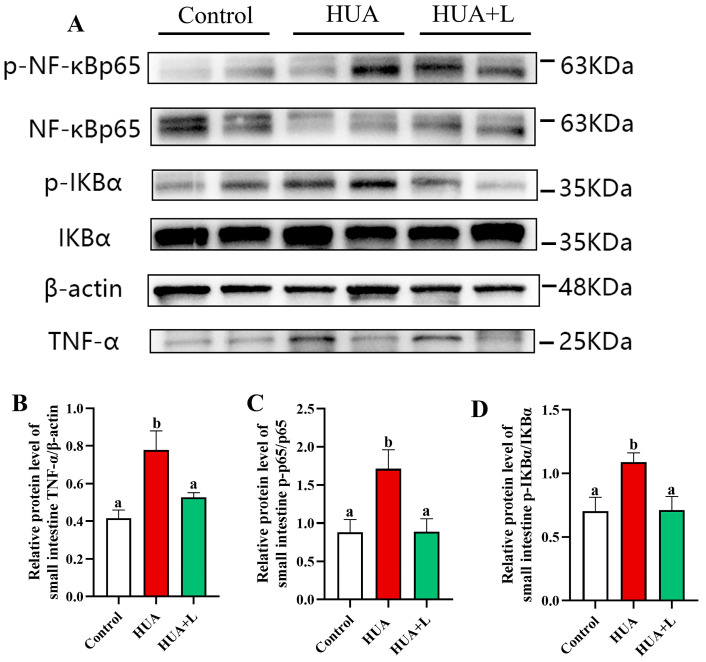
Effect of composite herbal tea on the NF-κB signaling pathway in the small intestine of hyperuricemic mice. (**A**) Western blot analysis of TNF-α, p-NF-κB p65, NF-κB p65, p -IκBα, and IκBα. (**B**) Relative protein expression of TNF-α, with target protein content normalized to β-actin. (**C**) Relative protein expression of p-NF-κB p65, with target protein content normalized to NF-κB p65. (**D**) Relative protein expression of p-IκBα, with target protein content normalized to IκBα. Data are expressed as mean ± SEM (*n* = 6). Different letters (a,b) indicate significant differences between groups for the same indicator (*p* < 0.05).

To elucidate the potential mechanism by which PHT ameliorates intestinal inflammation in hyperuricemic mice, we detected key proteins of the NF-κB signaling pathway, including TNF-α, p-NF-κB p65, NF-κB p65, p-IκBα, and IκBα, using Western blotting (Figure 4). The protein expression of TNF-α/β-actin was significantly increased by 87.3% in the HUA group compared with that in the control group (*p* < 0.05) and decreased by 18.9% in the HUA+L group. As shown in Figure 4C and D, the expression levels of p-NF-κB p65/NF-κB p65 and p-IκBα/IκBα in the HUA group were significantly higher than those in the control group, indicating that elevated uric acid activated the NF-κB signaling pathway. Notably, the expression levels of p-NF-κB p65 and p-IκBα were decreased by 48.2% and 34.7%, respectively, in the low-dose PHT-treated hyperuricemic mice compared with those in HUA mice. In conclusion, the low-dose PHT significantly blocked the overactivation of the NF-κB signaling pathway and significantly inhibited the expression of downstream inflammatory factor (TNF-α) proteins, thus exerting an anti-inflammatory effect at the intestinal level, which further supports its protective effect against intestinal inflammation in HUA mice.

### 3.5. PHT Modulated the Gut Microbiota in Hyperuricemic Mice

**Figure 5:**
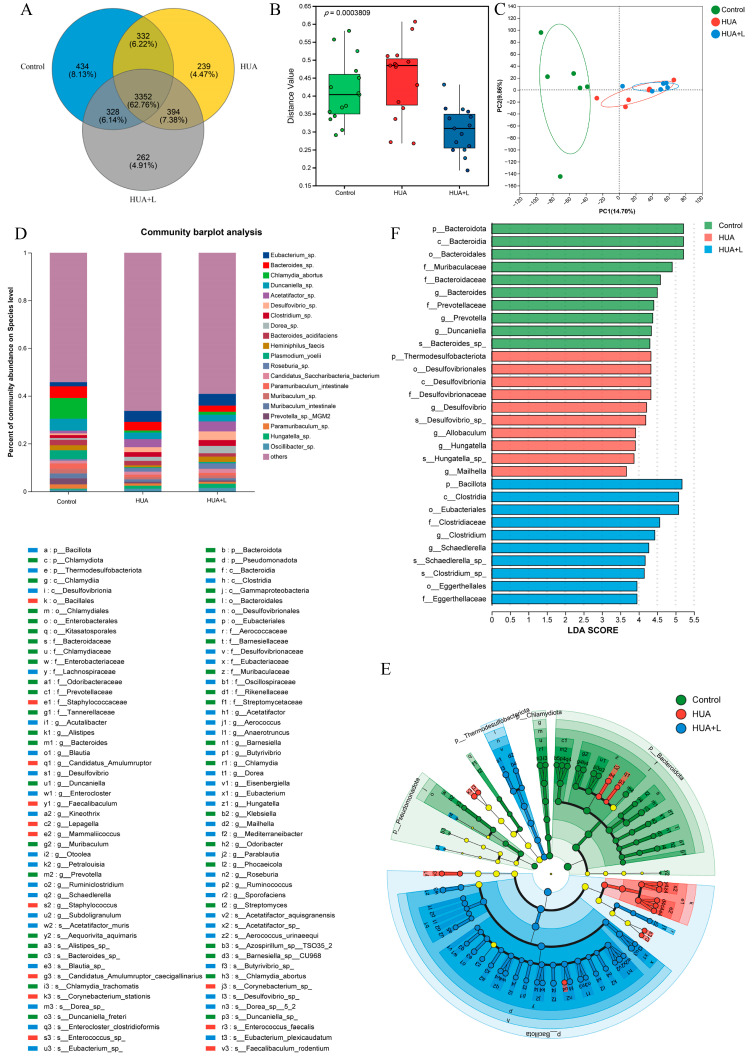
Effect of PHT on the gut microbiota of hyperuricemic mice. (**A**) Overlap of OTUs in the gut microbiota of each group of samples. (**B**) β-diversity box plot illustrating the diversity of each group. (**C**) Principal component (PCA) analysis plot. (**D**) Relative abundance of different groups of mouse gut microbiota at the species level. (**E**,**F**) LEfSe analysis (from phylum to species, LDA score > 3.0).

Information on species shared with and unique to the different groups was analyzed via a Venn diagram. The results are shown in Figure 5A, in which the number of OTUs shared by the three groups was 3352. The number of OTUs specific to the control group was 434, the number of OTUs specific to the HUA group was 239, and the number of OTUs specific to the HUA+L group was 262. The number of OTUs shared between the control and HUA groups was 332, and the number of OTUs shared by the HUA and HUA+L groups was 394. The unique species of the HUA+L group may be the key to the ability of PHT to reduce uric acid in hyperuricemic mice.

To further explore the effects of PHT on the gut microbiota in HUA mice, we analyzed the gut microbiota from the feces of the sacrificed mice. We assessed changes in the structure of the microbiota of each group by metagenome sequencing. Data from the alpha diversity analysis showed that the Shannon index (diversity index) and Chao index (richness index) did not differ significantly among the control, HUA, and HUA+L groups (Figure S1). However, PHT altered the β-diversity (Figure 5B), indicating that there were significant differences in the structure of the gut microbiota among the three groups of mice (*p* < 0.05). The results showed that the control, HUA, and HUA+L groups presented different gut microbiota compositions within their unique clusters (Figure 5C). More specifically, the microbial community structure of the HUA-treated group was very different from that of the control group but tended to diverge from that of the HUA group after the tea intervention. As seen from the species stacked histogram, at the species level, the percent abundance of strains *Eubacterium* sp., *Acetatifactor* sp., *Desulfovibrio* sp., *Clostridium* sp., *Dorea* sp., and Candidatus Saccharibacteria bacterium increased and the percent abundance of strains *Chlamydia abortus*, *Duncaniella* sp., *Heminiphilus faecis*, *Plasmodium yoelii*, *Paramuribaculum intestinale*, and *Muribaculum* sp. decreased in the HUA group compared with the control group. The tea intervention reversed this trend, resulting in a higher percent abundance of *Duncaniella* sp., *Heminiphilus faecis*, *Paramuribaculum intestinale*, and *Muribaculum* sp. Notably, the prevalence of *Heminiphilus faecis* was 2.05% in the control group and 1.29% lower in the HUA group. PHT reversed this trend, increasing the proportion of this bacteria by 1.53% in the HUA+L group.

The different bacteria enriched by the different interventions were screened via linear discriminant analysis effect size (LEfSe) analysis, which resulted in cladograms and distributions of the LDA values. Overall, we found that a total of 100 specific bacteria were identified from phylum to species (LDA > 3), with 40, 13, and 47 specific bacteria enriched in the control, HUA, and HUA+L groups, respectively (Figure 5E). Bacterial taxa with significant differences between the HUA group and HUA+L group were identified via LEfSe (LDA > 3) analysis. Bacillales (order), Staphylococcaceae (family), *Staphylococcus* (genus), *Mammaliicoccus* (genus), *Lepagella muris* sp., *Lepagella* (genus), *Corynebacterium stationis* sp., *Staphylococcus* sp., *Mammaliicoccus* sp., and *Mammaliicoccus sciuri* sp. were enriched in the HUA group, as shown in Figure 5F.

Clostridia (class), Eubacteriales (order), Bacillota (phylum), Lachnospiraceae (family), Thermodesulfobacteriota (phylum), Desulfovibrionales (order), Desulfovibrionia (class), Desulfovibrionaceae (family), *Desulfovibrio* (genus), and Eubacteriaceae (family) were enriched in the HUA+L group. The above results suggest that PHT intake altered the composition and diversity of the gut microbiota in the HUA mice.

### 3.6. PHT Functional Composition Analysis

To clarify the chemical basis of PHT and explore its potential biological activities, we first analyzed the functional components of PHT via UPLC–MS. A total of 2321 compounds were identified, of which 1269 were identified in positive ion mode and 1052 were identified in negative ion mode. Specific information on these compounds is provided in the Supplementary Materials. These compounds included polyphenols, saponins, anthraquinones, and other active substances (Table S1). All the compounds identified in PHT were annotated with compound classes according to the NPClassifier classification method [50]. The statistical results of the number of compounds identified for each pathway and major superclass are shown in Table S2. Shikimates and phenylpropanoids accounted for the largest share (543, 23.40%), followed by alkaloids (458, 19.73%), terpenoids (349, 15.04%), and others. Among the polyphenols, epicatechin gallate (ECG) and its derivatives, such as kaempferol, signaled most prominently. Among flavonoids, compounds such as isorhamnetin and luteolin have been reported to have potential anti-inflammatory, uric acid-lowering, and organ-protective functions [51,52]. Some anthraquinones (e.g., emodol and rhein) from cassia seeds have also been identified, and it has been hypothesized that they may facilitate intestinal excretory functions.

Notably, we also combined UPLC–MS analysis of cecum contents in follow-up experiments and found that some of the key compounds detected with PHT could still be present in their original form in the cecum. A total of 154 prototypical compounds were identified in the cecum contents of the mice in the HUA+L group: 49 in positive ionic mode and 105 in negative ionic mode (Table 2). Shikimates and phenylpropanoids accounted for the largest proportion of compounds (47, 30.52%), followed by alkaloids (25, 16.23%) and terpenoids (17, 11.04%). The compounds with prominent signals in negative ion mode were rhein, epicatechin gallate, vicenin-2, kaempferol, emodol, isorhamnetol, (-)-catechin, p-coumaric acid, luteolin, and (-)-quinic acid. These findings suggest that these functional components are not fully degraded or absorbed and may exert their biological activities directly in the intestine and participate in the regulation of uric acid metabolism and intestinal barrier function.

## 4. Discussion

In this study, we established a hyperuricemic mouse model induced by potassium oxonate (PO) and hypoxanthine (HX) to investigate the protective effects of PHT on intestinal uric acid excretion, barrier integrity, gut inflammation, and microbiota composition. This study primarily focused on evaluating PHT’s efficacy to mitigate intestinal injury associated with hyperuricemia and to provide new insights into its role as a protective functional food for hyperuricemia.

As a key enzyme in uric acid synthesis, XOD catalyzes the oxidation of hypoxanthine to xanthine and subsequently to uric acid [53]. We found that PHT significantly inhibited the hepatic XOD activity in HUA mice, thereby reducing the purine accumulation, which is consistent with the result of previous studies [54]. Additionally, PHT-treated mice exhibited a lower kidney index compared to HUA mice, suggesting a protective effect against HUA-induced renal hypertrophy. Although the kidneys are a primary site of uric acid excretion, this study specifically aimed to investigate PHT’s protective effects on intestinal injury associated with hyperuricemia. Prior research has indicated that HUA not only impairs renal function but also compromises intestinal barrier integrity and induces gut inflammation, while intestinal dysfunction itself can exacerbate HUA by impairing uric acid metabolism and excretion [55,56]. Given the emerging recognition of the intestine’s role in uric acid homeostasis [57], our study focused on elucidating the mechanisms through which PHT improves intestine injury in hyperuricemia. Furthermore, we evaluated PHT as a preventive intervention rather than a pharmacological treatment for HUA. Unlike conventional uric acid-lowering drugs, such as allopurinol and febuxostat, which primarily act by inhibiting XOD or enhancing renal uric acid excretion, PHT appears to exert its effects by modulating the gut microbiota, strengthening the intestinal barrier, and mitigating gut-derived inflammation. Consequently, direct comparisons between PHT and pharmacological treatments are not applicable. Instead, our findings suggest that PHT may serve as a functional food that supports gut health and alleviates intestinal dysfunction associated with hyperuricemia.

According to previous reports, PHT is a functional tea designed to relieve constipation [58]. The active ingredients in PHT—including anthraquinones from *Senna alexandrina* (e.g., sennosides A/B) and flavonoids from *Senna tora*—have demonstrated intestinal motility-enhancing effects [59]. These components may mechanistically contribute to the numerically reduced body weights in HUA+L/M/H groups versus controls despite the absence of statistical significance (Figure 1B). PHT significantly upregulated the mRNA expression levels of the intestinal uric acid transporter proteins ABCG2 and GLUT9 (Figure 1H,I). These proteins in the intestinal epithelium are responsible for transporting uric acid from the blood to the intestinal lumen, thus promoting uric acid excretion [60]. Similar findings have been reported in previous studies, where whey protein peptides enhanced intestinal uric acid excretion by upregulating ABCG2 and GLUT9 [61]. Notably, GLUT9 plays a crucial role in uric acid transport, as demonstrated in studies showing that GLUT9-deficient mice exhibit reduced fecal uric acid levels and an increased risk of metabolic disorders such as hypertension and dyslipidemia [62]. These findings underscore the significance of ABCG2 and GLUT9 in intestinal uric acid excretion and their potential contribution to reducing serum uric acid levels.

Intestinal barrier integrity is essential for gut homeostasis and uric acid metabolism. The complete intestinal barrier consists of a physical barrier (tight connections between cells), a chemical barrier (the mucosa), a biological barrier (gut microbiota), and an immune barrier (immune cells) [63,64]. In HUA mice, intestinal injury was evident, with villous atrophy, mucosal edema, and increased permeability, leading to higher serum LPS levels and inflammation (Figure 2A). These findings are consistent with previous findings demonstrating that elevated serum UA is associated with reduced intestinal barrier integrity [65,66]. However, PHT treatment significantly preserved intestinal structure, with enhanced expression of tight junction proteins (ZO-1, occludin) and mucins (MUC2), indicating restoration of gut integrity. Tight junction proteins occludin and ZO-1 play crucial roles in maintaining epithelial integrity and regulating intestinal permeability. Occludin, as a core backbone protein of tight junctions, stabilizes the barrier structure, while ZO-1 functions as a scaffold protein, linking occludin to the cytoskeleton [67]. Previous studies have shown that ZO-1 deficiency impairs mucosal healing, leading to barrier dysfunction and increased intestinal permeability in colitis models [68]. Similarly, MUC2, a key secreted mucin, constitutes the primary component of the mucus barrier and is critical for protecting the intestinal epithelium from bacterial invasion and inflammatory damage [69,70]. Emerging evidence further suggests that MUC2 not only reinforces barrier function but also influences microbiome composition and immune regulation [71]. In this study, HUA mice exhibited significant reductions in occludin, ZO-1, and MUC2 expression, consistent with barrier impairment and inflammation-induced damage. Notably, PHT reversed these changes, suggesting that its protective effects involve strengthening tight junctions, enhancing mucus secretion, and restoring barrier integrity. These findings indicate that PHT may prevent gut permeability through multiple pathways, ultimately reducing systemic inflammation and microbial disorder.

Disruption of the intestinal barrier leads to increased permeability and immune dysregulation, resulting in the translocation of bacterial endotoxins such as lipopolysaccharide (LPS) into the systemic circulation. This process triggers an inflammatory cascade characterized by elevated pro-inflammatory cytokines, which further exacerbate intestinal barrier dysfunction and gut dysbiosis [72]. Consistent with previous studies, we observed that HUA mice exhibited significantly higher serum LPS levels and increased expression of inflammatory markers (TNF-α and IL-6) in the intestine, along with a slight but non-significant elevation in IL-2 expression (Figure 3). These findings confirm that hyperuricemia-induced gut barrier impairment contributes to a heightened inflammatory response. Notably, PHT treatment effectively downregulated the expression of inflammatory cytokines, suggesting a protective role against gut-derived inflammation. Further mechanistic analysis revealed that PHT suppresses NF-κB signaling, a central pathway in inflammation regulation. Under inflammatory conditions, NF-κB activation is triggered by LPS and other proinflammatory stimuli, leading to the phosphorylation and degradation of IκBα and subsequent nuclear translocation of NF-κB p65, which drives the transcription of proinflammatory cytokines [73]. Our findings demonstrate that PHT mitigates this process, thereby reducing inflammation and preserving intestinal homeostasis.

Gut dysbiosis has been increasingly recognized as a contributing factor in hyperuricemia pathophysiology. In this study, we observed significant alterations in the gut microbiota composition of HUA mice, consistent with previous findings that hyperuricemia is associated with microbiota imbalances [74]. Notably, HUA mice exhibited an increased abundance of Gram-negative bacteria, particularly Desulfovibrio and Clostridium, both of which are linked to lipopolysaccharide (LPS) production, metabolic dysfunction, and intestinal inflammation [75,76,77]. Desulfovibrio, a member of Proteobacteria, has been implicated in diet-induced obesity and metabolic syndrome, with evidence suggesting that its hydrogen sulfide (H₂S) production may impair gut hormone signaling (GLP-1), thereby disrupting host metabolism [78]. These findings indicate that gut microbiota dysbiosis may exacerbate hyperuricemia through inflammation-driven metabolic disturbances. Conversely, beneficial bacterial genera such as Muribaculaceae (including Duncaniella, Paramuribaculum, and Muribaculum) were significantly reduced in HUA mice, but this trend was reversed by PHT treatment. Muribaculaceae play a key role in intestinal health and are major producers of short-chain fatty acids (SCFAs), which contribute to gut barrier integrity and inflammation regulation [79,80]. Previous studies have shown that Muribaculaceae enrichment is associated with improved gut health and metabolic regulation, with folic acid and fucoidan interventions increasing Muribaculaceae abundance and enhancing SCFA production (especially butyrate) [81,82]. Interestingly, PHT also increased the abundance of *Roseburia*, a well-known SCFA-producing bacterium that supports intestinal barrier integrity. In a study by Chen et al. [83], *Roseburia* abundance was enhanced following Kidney Tea intervention, suggesting that polyherbal formulations may exert gut-protective effects via microbiota modulation. However, further studies are needed to quantify SCFA production following PHT treatment and determine its role in hyperuricemia management.

To further elucidate the molecular basis of PHT’s effects, we conducted UPLC-MS analysis, which identified 154 prototypical compounds in the intestinal lumen following PHT treatment. These compounds had a synergistic effect, showing the potential to inhibit UA production and promote uric acid excretion in the gut. Among these 154 compounds with high signal responses were kaempferol, epicatechin gallate, (-)-catechin, isorhamnetol, luteolin, p-coumaric acid, D-sorbose, rhein, emodol, and xylose. Shikimates and phenylpropanoids accounted for the largest proportion of these compounds (Table 2). Notably, several phenylpropanoids, including isorhamnetol and luteolin, have been shown to inhibit XOD activity, thereby reducing serum uric acid levels and alleviating HUA [84,85,86]. These findings align with our results and suggest that PHT-derived bioactive compounds contribute to its protective effects.

## 5. Conclusions

In conclusion, PHT effectively prevents HUA, protects the intestinal barrier, modulates inflammation, and restores gut microbiota homeostasis in mice. The key mechanisms include (1) inhibition of XOD activity, reduction in uric acid production, and upregulation of intestinal UA transporter proteins (ABCG2, GLUT9), which promote UA excretion; (2) repair of the intestinal barrier through the regulation of physical–chemical barrier-related genes (occludin, ZO-1, and MUC2) and the suppression of inflammatory factors via NF-κB inhibition; and (3) modulation of the gut microbiota composition, which increases the number of beneficial bacteria to restore the intestinal balance. These findings suggest that PHT has a strong potential for preventing HUA. Our study provides an important theoretical basis for the development of functional foods with the potential to lower uric acid and protect intestinal function.

## Figures and Tables

**Table 1:** Primer sequences of RT-qPCR.

Gene	Forward Primer (5′→3′)	Reverse Primer (5′→3′)
β-Actin	TATGCTCTCCCTCACGCCATCC	GTCACGCACGATTTCCCTCTCAG
occludin	ACGGACCCTGACCACTATGA	TCAGCAGCAGCCATGTACTC
ZO-1	ACCCGAAACTGATGCTGTGGATAG	AAATGGCCGGGCAGAACTTGTGTA
MUC2	TGTGGTCTGTGTGGGAACTT	GCTTACATCTGGGCAAGTGG
GLUT9	TTGCTTTAGCTTCCCTGATGTG	GAGAGGTTGTACCCGTAGAGG
ABCG2	GGCCTGGACAAAGTAGCAGA	GTTGTGGGCTCATCCAGGAA
TNF-α	CCCTCACACTCAGATCATCTTCT	GCTACGACGTGGGCTACAG
IL-6	TAGTCCTTCCTACCCCAATTTCC	TTGGTCCTTAGCCACTCCTTC
IL-2	TGAGCAGGATGGAGAATTACAGG	GTCCAAGTTCATCTTCTAGGCAC

**Table 2:** Bioactive compounds in PHT persistent in the mouse gut.

Class	RT/min	Precursor *m*/*z*	Compound Name	Relative Content (%)	Adduct	Formula
Shikimates and phenylpropanoids (47, 30.52%)	1.94	225.07	(4-Acetyl-2-methoxyphenoxy)acetic acid	0.036	[M+H]^+^	C_11_H_12_O_5_
	6.93	315.08	Altenuene	0.421	[M+Na]^+^	C_15_H_16_O_6_
	3.65	317.12	4-Fluoro-2′,4′,6′-trimethoxychalcone	1.474	[M+H]^+^	C_18_H_17_FO_4_
	18.58	391.28	Dioctyl phthalate	1.258	[M+H]^+^	C_24_H_38_O_4_
	4.09	593.15	Vicenin-2	0.008	[M-H]^−^	C_27_H_30_O_15_
	5.03	639.16	2-(3,4-Dihydroxyphenyl)-5-hydroxy-7-methoxy-4-oxo-4H-chromen-3-yl 2-O-.beta.-D-glucopyranosyl-.beta.-D-glucopyranoside	0.004	[M-H]^−^	C_28_H_32_O_17_
	5.16	441.08	Epicatechin gallate	0.008	[M-H]^−^	C_22_H_18_O_10_
	8.86	285.04	Kaempferol	0.005	[M-H]^−^	C_15_H_10_O_6_
	9.16	315.05	Isorhamnetol	0.083	[M-H]^−^	C_16_H_12_O_7_
	3.36	125.03	Ganhuangemin	0.258	[M-H-C_9_H_6_O_4_]^−^	C_15_H_12_O_7_
	3.43	137.02	(-)-Catechin	0.012	[M-H-C_8_H_8_O_3_]^−^	C_15_H_14_O_6_
	2.61	151.04	2-Hydroxyphenylacetic acid	0.002	[M-H]^−^	C_8_H_8_O_3_
	1.87	151.04	4-Formyl-3-methoxyphenol	4.358	[M-H]^−^	C_8_H_8_O_3_
	3.13	151.04	2,5-Dihydroxyacetophenone	0.003	[M-H]^−^	C_8_H_8_O_3_
	4.36	153.02	Quercetagetinidin cation	0.061	[Cat-2H-C_8_H_4_O_3_]^−^	C_15_H_11_O_7_
	2.82	153.06	2-(2-Hydroxyethoxy)phenol	0.436	[M-H]^−^	C_8_H_10_O_3_
	3.57	163.04	2,4-Dihydroxy-3,6-dimethylbenzoic acid	0.032	[M-H-H_2_O]^−^	C_9_H_10_O_4_
	5.05	163.04	p-Coumaric acid	0.009	[M-H]^−^	C_9_H_8_O_3_
	3.58	165.02	3-Formyl-2-hydroxybenzoic acid	0.003	[M-H]^−^	C_8_H_6_O_4_
	5.15	169.02	3-Galloylgallocatechin	0.008	[M-H-C_15_H_12_O_6_]^−^	C_22_H_18_O_11_
	8.07	177.06	Methyl 4-coumarate	0.237	[M-H]^−^	C_10_H_10_O_3_
	4.97	179.04	5-Acetylsalicylic acid	0.005	[M-H]^−^	C_9_H_8_O_4_
	5.86	181.05	3,4-Dihydroxyhydrocinnamic acid	0.006	[M-H]^−^	C_9_H_10_O_4_
	7.39	181.05	Homovanillic acid	0.021	[M-H]^−^	C_9_H_10_O_4_
	1.53	189.04	3-Dehydroquinic acid	0.264	[M-H]^−^	C_7_H_10_O_6_
	3.42	193.05	3-(3,4-Dimethoxyphenyl)-2-hydroxy-3-methoxypropyl (2E)-3-(3,4-dihydroxyphenyl)prop-2-enoate	0.249	[M-H-C_11_H_14_O_4_]^−^	C_21_H_24_O_8_
	3.81	225.05	3-Hydroxy-4-methylbenzo[c]chromen-6-one	9.177	[M-H]^−^	C_14_H_10_O_3_
	7.72	227.07	Resveratrol	0.770	[M-H]^−^	C_14_H_12_O_3_
	10.71	267.07	7-Hydroxy-3-(4-hydroxyphenyl)-4-methylcoumarin	0.327	[M-H]^−^	C_16_H_12_O_4_
	9.89	283.06	5,7-dihydroxy-2′-methoxyflavone	0.084	[M-H]^−^	C_16_H_12_O_5_
	7.42	285.04	Luteolin	0.239	[M-H]^−^	C_15_H_10_O_6_
	3.48	299.08	Salicylic acid beta-D-glucoside	0.001	[M-H]^−^	C_13_H_16_O_8_
	10.02	300.03	N-(2-Hydroxyphenyl)-2,4,6-trinitrobenzamide	0.050	[M-H-HNO_2_]^−^	C_13_H_8_N_4_O_8_
	3.49	313.06	Salicyl glucuronide	0.033	[M-H]^−^	C_13_H_14_O_9_
	4.02	315.09	3-(3,4-Dimethoxyphenyl)-1-(2-hydroxy-4,6-dimethoxyphenyl)propan-1-one	1.722	[M-H-C_2_H_6_]^−^	C_19_H_22_O_6_
	11.10	327.09	3-Hydroxy-7,8,3′-trimethoxyflavone	3.306	[M-H]^−^	C_18_H_16_O_6_
	5.06	327.09	2,4-Bis(4-hydroxyphenyl)cyclobutane-1,3-dicarboxylic acid	0.008	[M-H]^−^	C_18_H_16_O_6_
	1.89	329.09	Helicid	0.785	[M+HCO_2_]^−^	C_13_H_16_O_7_
	11.45	337.04	6,7′-Dihydroxy-2,2’-dioxo-2H,2’H-[8,8’-bichromen]-7-yl hexopyranoside	0.008	[M-H-C_6_H_10_O_5_]^−^	C_24_H_20_O_12_
	5.84	417.08	Kaempferol 3-xyloside	0.341	[M-H]^−^	C_20_H_18_O_10_
	6.43	419.14	Rhapontin	0.003	[M-H]^−^	C_21_H_24_O_9_
	5.13	463.09	Hyperin	0.053	[M-H]^−^	C_21_H_20_O_12_
	6.25	473.17	2-(.beta.-D-Glucopyranosyloxy)-4-(prop-2-en-1-yl)phenyl .beta.-D-glucopyranoside	1.893	[M-H]^−^	C_21_H_30_O_12_
	4.12	609.15	Cyanin cation	0.166	[Cat-2H]^−^	C_27_H_31_O_16_
	4.31	635.09	1,3,6-Trigalloylglucose	0.037	[M-H]^−^	C_27_H_24_O_18_
	2.62	651.20	4-O-D-Glucopyranosyl-p-coumaric acid	0.891	[2M-H]^−^	C_15_H_18_O_8_
	2.44	665.06	Fenoxaprop	0.029	[2M-H]^−^	C_16_H_12_ClNO_5_
Alkaloids (25, 16.23%)	1.15	110.06	3-Cyanocyclopentanone	1.461	[M+H]^+^	C_6_H_7_NO
	0.86	136.06	Adenine	0.266	[M+H]^+^	C_5_H_5_N_5_
	1.88	152.07	Adrenochrome	0.346	[M+H-CO]^+^	C_9_H_9_NO_3_
	1.66	166.12	(-)-Pseudoephedrine	0.007	[M+H]^+^	C_10_H_15_NO
	1.14	169.03	Uric acid	0.006	[M+H]^+^	C_5_H_4_N_4_O_3_
	4.01	178.09	1-(o-Hydroxyphenyl)-2-pyrrolidinone	0.304	[M+H]^+^	C_10_H_11_NO_2_
	1.52	203.08	2,3-Dimethyl-6-quinoxalinecarboxylic acid	4.466	[M+H]^+^	C_11_H_10_N_2_O_2_
	2.24	220.13	Ritalinic acid	0.189	[M+H]^+^	C_13_H_17_NO_2_
	9.92	229.08	Galanthaminone	0.022	[M+H-C_3_H_7_N]^+^	C_17_H_19_NO_3_
	3.18	237.16	(.+/-.)-Dropropizine	0.977	[M+H]^+^	C_13_H_20_N_2_O_2_
	2.62	255.13	3-(3,4,5-Trimethoxyphenyl)propanohydrazide	0.002	[M+H]^+^	C_12_H_18_N_2_O_4_
	0.83	260.11	1-Piperazineethanol, 4-(7-nitro-4-benzofurazanyl)-	0.637	[M+H-H_2_O_2_]^+^	C_12_H_15_N_5_O_4_
	2.85	144.05	7-Quinolinol	0.150	[M-H]^−^	C_9_H_7_NO
	2.64	155.05	1,3-Dimethylbarbituric acid	0.045	[M-H]^−^	C_6_H_8_N_2_O_3_
	5.02	160.04	4-[(E)-6,7-Dihydroxy-3,7-dimethyl-oct-2-enoxy]carbostyril	6.548	[M-H-C_10_H_18_O_2_]^−^	C_19_H_25_NO_4_
	4.72	190.05	5-Hydroxyindole-3-acetic acid	0.032	[M-H]^−^	C_10_H_9_NO_3_
	3.53	193.10	4-(Methylamino)-4-(3-pyridyl)butyric acid	0.022	[M-H]^−^	C_10_H_14_N_2_O_2_
	6.67	213.11	Sulfisomidin	2.936	[M-H-SO_2_]^−^	C_12_H_14_N_4_O_2_S
	4.01	227.06	1-Phenyl-1H-pyrazolo[3,4-d]pyrimidine-4,6-diol	0.536	[M-H]^−^	C_11_H_8_N_4_O_2_
	14.84	250.15	2-[(2-Ethylbutanoyl)amino]-4,5-dimethoxybenzoic acid	0.104	[M-H-CO_2_]^−^	C_15_H_21_NO_5_
	2.34	275.11	Ofloxacin	2.422	[M-H-C_4_H_5_O_2_]^−^	C_18_H_20_FN_3_O_4_
	1.48	279.07	N-(1,3-Dioxo-2,3-dihydro-1H-isoindol-5-yl)-2-phenylacetamide	0.022	[M-H]^−^	C_16_H_12_N_2_O_3_
	6.09	417.20	Dauricine	0.110	[M-H-C_12_H_16_NO_2_]^−^	C_38_H_44_N_2_O_6_
	7.19	449.15	3-(3,4-Dihydroxy-1-keto-2,2-dimethyl-3aH-imidaz[1,2-a]indol-4-yl)-2-(4-ketoquinazolin-3-yl)propionic acid	0.784	[M-H]^−^	C_23_H_22_N_4_O_6_
	2.66	527.25	Chaetoglobosin A	1.321	[M-H]^−^	C_32_H_36_N_2_O_5_
Terpenoids (17, 11.04%)	9.15	180.17	Rimantadine	0.433	[M+H]^+^	C_12_H_21_N
	10.84	185.13	Parthenolide	1.336	[M+H-CH_4_O_3_]^+^	C_15_H_20_O_3_
	9.43	247.13	Bisbynin	0.096	[M+H-2H_2_O]^+^	C_15_H_22_O_5_
	11.87	261.18	Jaeskeanadiol	0.001	[M+Na]^+^	C_15_H_26_O_2_
	9.69	367.19	Uabanin	0.006	[M+H-C_6_H_18_O_8_]^+^	C_29_H_44_O_12_
	9.76	385.24	Resinobufagin	4.430	[M+H]^+^	C_24_H_32_O_4_
	3.73	201.08	3-(2-Hydroxybutan-2-yl)-5-oxooxolane-3-carboxylic acid	0.025	[M-H]^−^	C_9_H_14_O_5_
	6.43	203.14	Viscic acid	0.002	[M-H-CH_2_O_2_]^−^	C_15_H_22_O_3_
	6.43	265.15	Cyperanic acid	0.749	[M-H]^−^	C_15_H_22_O_4_
	8.39	267.16	2-((1S,2S,4aR,8aS)-1-hydroxy-4a-methyl-8-methylenedecahydronaphthalen-2-yl)acrylic acid	0.019	[M+OH]^−^	C_15_H_22_O_3_
	1.76	271.13	2,6-Dihydroxy-1,1,7-trimethyl-2,9,10,10a-tetrahydrophenanthren-3-one	0.089	[M-H]^−^	C_17_H_20_O_3_
	11.67	287.17	4-(4-Hydroxyphenyl)-6,8,9-trimethyl-3-oxabicyclo[3.3.1]non-6-ene-1-methanol	0.265	[M-H]^−^	C_18_H_24_O_3_
	7.38	357.16	9-Hydroxyjasmesoside	0.005	[M-H-C_10_H_16_O_6_]^−^	C_27_H_42_O_14_
	1.88	371.10	11-Methyloleoside	0.030	[M-H-CH_4_O]^−^	C_17_H_24_O_11_
	4.36	445.23	[7′-Formyl-3,4′-dihydroxy-6′-(hydroxymethyl)-4,4,7,8a-tetramethylspiro[2,3,4a,5,6,7-hexahydro-1H-naphthalene-8,2′-3H-1-benzofuran]-2-yl] acetate	0.012	[M-H]^−^	C_25_H_34_O_7_
	3.66	447.19	17.beta.-Estradiol 3-.beta.-D-glucuronide	0.033	[M-H]^−^	C_24_H_32_O_8_
	5.72	517.19	Gossypol	0.406	[M-H]^−^	C_30_H_30_O_8_
	10.72	517.32	Trachelosperogenin A1	0.006	[M-H-C_6_H_10_O_5_]^−^	C_36_H_56_O_12_

## Data Availability

The original contributions presented in this study are included in the article/Supplementary Materials. Further inquiries can be directed to the corresponding authors.

## Supplementary Materials

The following supporting information can be downloaded at: https://www.mdpi.com/article/10.3390/foods14071118/s1. Figure S1: Alpha diversity box plot analysis. (A) Ace. (B) Chao. (C) Shamon. (D) Sobs. (E) Simpson index; Table S1: PHT components were identified by UPLC-MS; Table S2: Ingredients in PHT were classified according to the NPClassifier method.

## References

[1] Mehmood A., Iftikhar A., Chen X. (2024). Food-derived bioactive peptides with anti-hyperuricemic activity: A comprehensive review. Food Chem..

[2] Du L., Zong Y., Li H., Wang Q., Xie L., Yang B., Pang Y., Zhang C., Zhong Z., Gao J. (2024). Hyperuricemia and its related diseases: Mechanisms and advances in therapy. Signal Transduct. Target. Ther..

[3] Pathmanathan K., Robinson P.C., Hill C.L., Keen H.I. (2021). The prevalence of gout and hyperuricaemia in Australia: An updated systematic review. Semin. Arthritis Rheum..

[4] Zhang M., Zhu X., Wu J., Huang Z., Zhao Z., Zhang X., Xue Y., Wan W., Li C., Zhang W. (2022). Prevalence of Hyperuricemia Among Chinese Adults: Findings From Two Nationally Representative Cross-Sectional Surveys in 2015–16 and 2018–19. Front. Immunol..

[5] Kim Y.J., Kim S., Seo J.H., Cho S.K. (2024). Prevalence and Associations Between Metabolic Syndrome Components and Hyperuricemia by Race: Findings From US Population, 2011–2020. Arthritis Care Res..

[6] Basnet T.B., Du S., Feng R., Gao J., Gong J., Ye W. (2023). Fatty liver mediates the association of hyperuricemia with prediabetes and diabetes: A weighting-based mediation analysis. Front. Endocrinol..

[7] Gu T., Cao G., Luo M., Zhang N., Xue T., Hou R., Leng M. (2022). A systematic review and meta-analysis of the hyperuricemia risk from certain metals. Clin. Rheumatol..

[8] Yu Y., Quan X., Wang H., Zhang B., Hou Y., Su C. (2023). Assessing the health risk of hyperuricemia in participants with persistent organic pollutants exposure—A systematic review and meta-analysis. Ecotox. Environ. Safe.

[9] Lv Q., Zhou J., Wang C., Yang X., Han Y., Zhou Q., Yao R., Sui A. (2023). A dynamics association study of gut barrier and microbiota in hyperuricemia. Front. Microbiol..

[10] Liu X., Lv Q., Ren H., Gao L., Zhao P., Yang X., Yang G., Xu D., Wang G., Yang W. (2020). The altered gut microbiota of high-purine-induced hyperuricemia rats and its correlation with hyperuricemia. Peerj.

[11] Ge H., Jiang Z., Li B., Xu P., Wu H., He X., Xu W., Huang Z., Xiong T., Wang P. (2023). Dendrobium officinalis Six Nostrum Promotes Intestinal Urate Underexcretion via Regulations of Urate Transporter Proteins in Hyperuricemic Rats. Comb. Chem. High Throughput Screen..

[12] Morimoto C., Tamura Y., Asakawa S., Kuribayashi-Okuma E., Nemoto Y., Li J., Murase T., Nakamura T., Hosoyamada M., Uchida S. (2020). ABCG2 expression and uric acid metabolism of the intestine in hyperuricemia model rat. Nucleosides Nucleotides Nucleic Acids.

[13] Yao W., Zhang Y., Zhang W., Wen Y., Yang R., Dong J., Zhang X., Hua Y., Ji P., Wei Y. (2022). Pathological mechanism of intestinal mucosal barrier injury of large intestine dampness-heat syndrome rats and the protective effect of Yujin powder. Res. Vet. Sci..

[14] Dong L., Luo P., Zhang A. (2024). Intestinal microbiota dysbiosis contributes to the liver damage in subchronic arsenic-exposed mice. Acta Biochim. Biophys. Sin..

[15] Yuan L., Liu C., Li B., Wang S., Sun J., Mao X. (2024). Multi-omics analysis reveals that agaro-oligosaccharides with different degrees of polymerization alleviate colitis in mice by regulating intestinal flora and arginine synthesis. Food Funct..

[16] Liu Z., Zhao J., Sun R., Wang M., Wang K., Li Y., Shang H., Hou J., Jiang Z. (2022). *Lactobacillus plantarum* 23-1 improves intestinal inflammation and barrier function through the TLR4/NF-κB signaling pathway in obese mice. Food Funct..

[17] Zhong J., Qiu X., Yu Q., Chen H., Yan C. (2020). A novel polysaccharide from *Acorus tatarinowii* protects against LPS-induced neuroinflammation and neurotoxicity by inhibiting TLR4-mediated MyD88/NF-κB and PI3K/Akt signaling pathways. Int. J. Biol. Macromol..

[18] Chen N., Wang R., Li H., Wang W., Wang L., Yin X., Yao R., Yang B. (2022). Flavonoid extract of saffron by-product alleviates hyperuricemia via inhibiting xanthine oxidase and modulating gut microbiota. Phytother. Res..

[19] Chu Y., Sun S., Huang Y., Gao Q., Xie X., Wang P., Li J., Liang L., He X., Jiang Y. (2021). Metagenomic analysis revealed the potential role of gut microbiome in gout. Npj Biofilms Microbomes.

[20] Shi R., Ye J., Fan H., Xiao C., Wang D., Xia B., Zhao Z., Zhao B., Dai X., Liu X. (2023). *Lactobacillus plantarum* LLY-606 supplementation ameliorates hyperuricemia via modulating intestinal homeostasis and relieving inflammation. Food Funct..

[21] Zou Y., Ro K., Jiang C., Yin D., Zhao L., Zhang D., Du L., Xie J. (2024). The anti-hyperuricemic and gut microbiota regulatory effects of a novel purine assimilatory strain, *Lactiplantibacillus plantarum* X7022. Eur. J. Nutr..

[22] Lee Y., Kim N., Werlinger P., Suh D., Lee H., Cho J., Cheng J. (2022). Probiotic Characterization of *Lactobacillus brevis* MJM60390 and In Vivo Assessment of Its Antihyperuricemic Activity. J. Med. Food.

[23] Li Y., Zhu J., Lin G., Gao K., Yu Y., Chen S., Chen L., Chen Z., Li L. (2022). Probiotic effects of *Lacticaseibacillus rhamnosus* 1155 and *Limosilactobacillus fermentum* 2644 on hyperuricemic rats. Front. Nutr..

[24] Wang J., Chen Y., Zhong H., Chen F., Regenstein J., Hu X., Cai L., Feng F. (2022). The gut microbiota as a target to control hyperuricemia pathogenesis: Potential mechanisms and therapeutic strategies. Crit. Rev. Food Sci. Nutr..

[25] Hutchinson A.N., Tingö L., Brummer R.J. (2020). The Potential Effects of Probiotics and ω-3 Fatty Acids on Chronic Low-Grade Inflammation. Nutrients.

[26] Xu L., Lu L.L., Gao J.D. (2021). Traditional Chinese Herbal Medicine Plays a Role in the Liver, Kidney, and Intestine to Ameliorate Hyperuricemia according to Experimental Studies. Evid.-Based Complement Altern. Med..

[27] Huang C., Chen T., Tsai G. (2022). Hypouricemic Effect of Submerged Culture of Ganoderma lucidum in Potassium Oxonate-Induced Hyperuricemic Rats. Metabolites.

[28] Wu Y., Wang Y., Ou J., Wan Q., Shi L., Li Y., He F., Wang H., He L., Gao J. (2018). Effect and Mechanism of ShiZhiFang on Uric Acid Metabolism in Hyperuricemic Rats. Evid.-Based Complement Altern. Med..

[29] Meng J., Tian J., Zhao Y., Li C., Yi Y., Zhang Y., Han J., Wang L., Pan C., Liu S. (2023). Ameliorative effect of cheqianzi decoction on hyperuricemia and kidney injury and underlying mechanism in rats. Heliyon.

[30] Wang Z., Liu J., Mou Y., Liao W., Li Y., Liu J., Tang J. (2024). Anti-inflammatory and uric acid lowering effects of Euodiae fructus on hyperuricemia and gout mice. Front. Pharmacol..

[31] Chen G., Tan M., Li K., Leung P., Ko C. (2015). Green tea polyphenols decrease uric acid level through xanthine oxidase and renal urate transporters in hyperuricemic mice. J. Ethnopharmacol..

[32] Zhu C., Xu Y., Liu Z., Wan X., Li D., Tai L. (2018). The anti-hyperuricemic effect of epigallocatechin-3-gallate (EGCG) on hyperuricemic mice. Biomed. Pharmacother..

[33] Huang L., Deng J., Chen G., Zhou M., Liang J., Yan B., Shu J., Liang Y., Huang H. (2019). The anti-hyperuricemic effect of four astilbin stereoisomers in Smilax glabra on hyperuricemic mice. J. Ethnopharmacol..

[34] Liang D., Yong T., Diao X., Chen S., Chen D., Xiao C., Zuo D., Xie Y., Zhou X., Hu H. (2021). Hypouricaemic and nephroprotective effects of Poria cocos in hyperuricemic mice by up-regulating ATP-binding cassette super-family G member 2. Pharm. Biol..

[35] Zhu L., Dong Y., Na S., Han R., Wei C., Chen G. (2017). Saponins extracted from Dioscorea collettii rhizomes regulate the expression of urate transporters in chronic hyperuricemia rats. Biomed. Pharmacother..

[36] Hou S., Chen S., Shen J., Chen H., Wang S., Wang C., Man K., Liu P., Tsai M., Chen Y. (2023). Emodin, a Natural Anthraquinone, Increases Uric Acid Excretion in Rats with Potassium Oxonate-Induced Hyperuricemia. Pharmaceuticals.

[37] Wu H., Zhou M., Lu G., Yang Z., Ji H., Hu Q. (2017). Emodinol ameliorates urate nephropathy by regulating renal organic ion transporters and inhibiting immune inflammatory responses in rats. Biomed. Pharmacother..

[38] Wang H., Wang C., Guo S., Chen Z., Peng Z., Duan R., Dong T.T.X., Tsim K.W.K. (2019). Polysaccharide deriving from Ophiopogonis Radix promotes metabolism of ginsenosides in the present of human gut microbiota based on UPLC-MS/MS assay. J. Pharm. Biomed. Anal..

[39] Wei X., Liang J., Liu J., Dai Y., Leng X., Cheng Y., Chi L. (2024). Anchang Yuyang Decoction inhibits experimental colitis-related carcinogenesis by regulating PPAR signaling pathway and affecting metabolic homeostasis of host and microbiota. J. Ethnopharmacol..

[40] Xu S.Y., Bian R.L., Chen X. (1982). Pharmacological Experimental Methodology.

[41] Li X., Gao X., Zhang H., Liu Y., Sarker M.M.R., Wu Y., Chen X., Zhao C. (2021). The anti-hyperuricemic effects of green alga Enteromorpha prolifera polysaccharide via regulation of the uric acid transporters in vivo. Food Chem. Toxicol..

[42] Xie D., Shen Y., Su E., Du L., Xie J., Wei D. (2023). Anti-Hyperuricemic, Nephroprotective, and Gut Microbiota Regulative Effects of Separated Hydrolysate of α-Lactalbumin on Potassium Oxonate- and Hypoxanthine-Induced Hyperuricemic Mice. Mol. Nutr. Food Res..

[43] Shen L., Yang Y., Zhang J., Feng L., Zhou Q. (2023). Diacylated anthocyanins from purple sweet potato (*Ipomoea batatas* L.) attenuate hyperglycemia and hyperuricemia in mice induced by a high-fructose/high-fat diet. J. Zhejiang Univ.-Sci. B.

[44] Chiu C., McArdle A.H., Brown R., Scott H.J., Gurd F.N. (1970). Intestinal Mucosal Lesion in Low-Flow States: I. A Morphological, Hemodynamic, and Metabolic Reappraisal. Arch. Surg..

[45] Ala M., Fallahpour Khoshdel M.R., Mohammad Jafari R., Sadrkhanloo M., Goudarzi S., Asl Soleimani M., Dehpour A.R. (2023). Low-dose sumatriptan improves the outcome of acute mesenteric ischemia in rats via downregulating kynurenine. Pharmacol. Rep..

[46] Jang Y.J., Kim W., Han D.H., Lee K., Ko G. (2019). *Lactobacillus fermentum* species ameliorate dextran sulfate sodium-induced colitis by regulating the immune response and altering gut microbiota. Gut Microbes.

[47] Okumura R., Takeda K. (2024). The role of the mucosal barrier system in maintaining gut symbiosis to prevent intestinal inflammation. Semin. Immunopathol..

[48] Li J., Zhang R., Ma J., Tang S., Li Y., Li Y., Wan J. (2021). Mucosa-Associated Microbial Profile Is Altered in Small Intestinal Bacterial Overgrowth. Front. Microbiol..

[49] Sun Y., Wu D., Zeng W., Chen Y., Guo M., Lu B., Li H., Sun C., Yang L., Jiang X. (2021). The Role of Intestinal Dysbacteriosis Induced Arachidonic Acid Metabolism Disorder in Inflammaging in Atherosclerosis. Front. Cell. Infect. Microbiol..

[50] Kim H.W., Wang M., Leber C.A., Nothias L., Reher R., Kang K.B., van der Hooft J.J.J., Dorrestein P.C., Gerwick W.H., Cottrell G.W. (2021). NPClassifier: A Deep Neural Network-Based Structural Classification Tool for Natural Products. J. Nat. Prod..

[51] Adachi S., Oyama M., Kondo S., Yagasaki K. (2021). Comparative effects of quercetin, luteolin, apigenin and their related polyphenols on uric acid production in cultured hepatocytes and suppression of purine bodies-induced hyperuricemia by rutin in mice. Cytotechnology.

[52] Wang F., Zhao X., Su X., Song D., Zou F., Fang L. (2021). Isorhamnetin, the xanthine oxidase inhibitor from Sophora japonica, ameliorates uric acid levels and renal function in hyperuricemic mice. Food Funct..

[53] Liu Y., Lu H., Fang Z., Lu S. (2025). Hesperetin acts as a potent xanthine oxidase inhibitor: New evidence from its reactive oxygen suppression and enzyme binding. Int. J. Biol. Macromol..

[54] Xiang L., Huang Y., Li R., Tao Y., Wu T., Pan S., Xu X. (2023). Artemisia selengensis Turcz. leaves extract ameliorates hyperuricemia in mice by inhibiting hepatic xanthine oxidase activity, modulating renal uric acid transporters, and improving metabolic disorders. Food Biosci..

[55] Li Y., Li H., Wang R., Yu Y., Liu X., Tian Z. (2023). Protective effect of sodium butyrate on intestinal barrier damage and uric acid reduction in hyperuricemia mice. Biomed. Pharmacother..

[56] Wang Z., Wu G., Niu T., Guo Y., Wang C., Wang X., Yu J. (2024). Polysaccharide isolated from Dioscorea septemloba improves hyperuricemia and alleviates renal fibrosis through gut-kidney axis in mice. Int. J. Biol. Macromol..

[57] Lv Q., Xu D., Zhang X., Yang X., Zhao P., Cui X., Liu X., Yang W., Yang G., Xing S. (2020). Association of Hyperuricemia With Immune Disorders and Intestinal Barrier Dysfunction. Front. Physiol..

[58] Fei W., Zhang J., Wang L., Yang Y., Chen Y., Chen Y., Tao R., Zhu Y. (2022). A clinical study to observe the efficacy and safety of Besunyen Detox Tea for constipation. Medicine.

[59] Ma Q., Wang C., Sawadogo W.R., Bian Z., Yuan C. (2022). Herbal Medicines for Constipation and Phytochemical Comparison of Active Components. Am. J. Chin. Med..

[60] Xu X., Li C., Zhou P., Jiang T. (2016). Uric acid transporters hiding in the intestine. Pharm. Biol..

[61] Qi X., Ma Y., Guan K., Zhao L., Ma Y., Wang R. (2024). Whey Protein Peptide Pro-Glu-Trp Ameliorates Hyperuricemia by Enhancing Intestinal Uric Acid Excretion, Modulating the Gut Microbiota, and Protecting the Intestinal Barrier in Rats. J. Agric. Food Chem..

[62] DeBosch B.J., Kluth O., Fujiwara H., Schürmann A., Moley K. (2014). Early-onset metabolic syndrome in mice lacking the intestinal uric acid transporter SLC2A9. Nat. Commun..

[63] Lee Y., Kamada N., Moon J.J. (2021). Oral nanomedicine for modulating immunity, intestinal barrier functions, and gut microbiome. Adv. Drug Deliv. Rev..

[64] Meng W., Chen L., Ouyang K., Lin S., Zhang Y., He J., Wang W. (2023). Chimonanthus nitens Oliv. leaves flavonoids alleviate hyperuricemia by regulating uric acid metabolism and intestinal homeostasis in mice. Food Sci. Hum. Wellness.

[65] Liu C., Ruan F., Chen Z., Han J., Ding X., Han C., Ye L., Yang C., Yu Y., Zuo Z. (2024). Phenanthrene-induced hyperuricemia with intestinal barrier damage and the protective role of theabrownin: Modulation by gut microbiota-mediated bile acid metabolism. Sci. Total Environ..

[66] Wang Q., Liang J., Zou Q., Wang W., Yan G., Guo R., Yuan T., Wang Y., Liu X., Liu Z. (2024). Tryptophan Metabolism-Regulating Probiotics Alleviate Hyperuricemia by Protecting the Gut Barrier Integrity and Enhancing Colonic Uric Acid Excretion. J. Agric. Food Chem..

[67] Liu X., Lin C., Dreffs A.A., Su E.J., Lawrence D.A., Antonetti D.A. (2024). Occludin carboxy terminus is a dynein adaptor required for mouse embryonic development. Investig. Ophthalmol. Vis. Sci..

[68] Kuo W., Zuo L., Odenwald M.A., Madha S., Singh G., Gurniak C.B., Abraham C., Turner J.R. (2021). The Tight Junction Protein ZO-1 Is Dispensable for Barrier Function but Critical for Effective Mucosal Repair. Gastroenterology.

[69] Yao D., Dai W., Dong M., Dai C., Wu S. (2021). MUC2 and related bacterial factors: Therapeutic targets for ulcerative colitis. Ebiomedicine.

[70] Hansson G.C. (2020). Mucins and the Microbiome. Annu. Rev. Biochem..

[71] Liu Y., Yu X., Zhao J., Zhang H., Zhai Q., Chen W. (2020). The role of MUC2 mucin in intestinal homeostasis and the impact of dietary components on MUC2 expression. Int. J. Biol. Macromol..

[72] Zhou Y., Zhao M., Pu Z., Xu G., Li X. (2018). Relationship between oxidative stress and inflammation in hyperuricemia: Analysis based on asymptomatic young patients with primary hyperuricemia. Medicine.

[73] He J., Han S., Li X., Wang Q., Cui Y., Chen Y., Gao H., Huang L., Yang S. (2019). Diethyl Blechnic Exhibits Anti-Inflammatory and Antioxidative Activity via the TLR4/MyD88 Signaling Pathway in LPS-Stimulated RAW264.7 Cells. Molecules.

[74] Zhu C., Niu H., Bian M., Zhang X., Zhang X., Zhou Z. (2023). Study on the mechanism of *Orthosiphon aristatus* (Blume) Miq. in the treatment of hyperuricemia by microbiome combined with metabonomics. J. Ethnopharmacol..

[75] Lakhal R., Pradel N., Postec A., Hamdi M., Ollivier B., Godfroy A., Fardeau M. (2013). *Vallitaleaguaymasensis* gen. nov., sp. nov., isolated from marine sediment. Int. J. Syst. Evol. Microbiol..

[76] Morotomi M., Nagai F., Watanabe Y. (2012). Description of *Christensenella minuta* gen. nov., sp. nov., isolated from human faeces, which forms a distinct branch in the order Clostridiales, and proposal of *Christensenellaceae* fam. nov. Int. J. Syst. Evol. Microbiol..

[77] Yoon J., Kang S., Park S., Lee S., Oh T. (2007). Reclassification of *Aquaspirillum itersonii* and *Aquaspirillum peregrinum* as *Novispirillum itersonii* gen. nov., comb. nov. and *Insolitispirillum peregrinum* gen. nov., comb. nov. Int. J. Syst. Evol. Microbiol..

[78] Qi Q., Zhang H., Jin Z., Wang C., Xia M., Chen B., Lv B., Peres Diaz L., Li X., Feng R. (2024). Hydrogen sulfide produced by the gut microbiota impairs host metabolism via reducing GLP-1 levels in male mice. Nat. Metab..

[79] Lagkouvardos I., Lesker T.R., Hitch T.C.A., Gálvez E.J.C., Smit N., Neuhaus K., Wang J., Baines J.F., Abt B., Stecher B. (2019). Sequence and cultivation study of Muribaculaceae reveals novel species, host preference, and functional potential of this yet undescribed family. Microbiome.

[80] Lagkouvardos I., Pukall R., Abt B., Foesel B.U., Meier-Kolthoff J.P., Kumar N., Bresciani A., Martínez I., Just S., Ziegler C. (2016). The Mouse Intestinal Bacterial Collection (miBC) provides host-specific insight into cultured diversity and functional potential of the gut microbiota. Nat. Microbiol..

[81] Wang P., Zhang X., Zheng X., Gao J., Shang M., Xu J., Liang H. (2022). Folic Acid Protects against Hyperuricemia in C57BL/6J Mice via Ameliorating Gut–Kidney Axis Dysfunction. J. Agric. Food Chem..

[82] Liu X., Zhang Y., Li W., Zhang B., Yin J., Liuqi S., Wang J., Peng B., Wang S. (2022). Fucoidan Ameliorated Dextran Sulfate Sodium-Induced Ulcerative Colitis by Modulating Gut Microbiota and Bile Acid Metabolism. J. Agric. Food Chem..

[83] Chen Y., Pei C., Chen Y., Xiao X., Zhang X., Cai K., Deng S., Liang R., Xie Z., Li P. (2023). Kidney tea ameliorates hyperuricemia in mice via altering gut microbiota and restoring metabolic profile. Chem.-Biol. Interact..

[84] Adachi S., Kondo S., Sato Y., Yoshizawa F., Yagasaki K. (2019). Anti-hyperuricemic effect of isorhamnetin in cultured hepatocytes and model mice: Structure–activity relationships of methylquercetins as inhibitors of uric acid production. Cytotechnology.

[85] Wang X., Zhao J., Lin Z., Li J., Li X., Xu X., Wang Y., Lv G., Lin H., Lin Z. (2024). Analysis of Polyphenol Extract from Hazel Leaf and Ameliorative Efficacy and Mechanism against Hyperuricemia Zebrafish Model via Network Pharmacology and Molecular Docking. Molecules.

[86] Yao J., He H., Xue J., Wang J., Jin H., Wu J., Hu J., Wang R., Kuchta K. (2019). Mori Ramulus (Chin.Ph.)—The Dried Twigs of Morus alba L./Part 1: Discovery of Two Novel Coumarin Glycosides from the Anti-Hyperuricemic Ethanol Extract. Molecules.

