# Supplementary material for: Protective Effects of a Polyherbal Mixture on Intestinal Injury via the NF-κB Signaling Pathway and Gut Microbiota Modulation in Hyperuricemic Mice
Source: Foods. 2025 Mar 24;14(7):1118. doi: 10.3390/foods14071118 (PMC11988963; doi:10.3390/foods14071118)
Supplement: Supplementary file 1 [file foods-14-01118-s001.zip › Supplementary Table S2 and Figure S1.pdf]

## Supplementary Tables

**Supplementary Table S2** Ingredients in PHT were classified according to the NPClassifier method.

| Pathway | SuperClass | Class | count |
|---|---|---|---|
| Alkaloids | Anthranilic acid alkaloids | Anthranillic acid derivatives | 4 |
| Alkaloids | Anthranilic acid alkaloids | Phenazine alkaloids | 4 |
| Alkaloids | Anthranilic acid alkaloids | Quinazoline alkaloids | 14 |
| Alkaloids | Anthranilic acid alkaloids | other | 26 |
| Alkaloids | Lysine alkaloids | Piperidine alkaloids | 18 |
| Alkaloids | Lysine alkaloids | other | 20 |
| Alkaloids | Pseudoalkaloids | Phenylalanine-derived alkaloids | 11 |
| Alkaloids | Pseudoalkaloids | Purine alkaloids | 32 |
| Alkaloids | Pseudoalkaloids | other | 19 |
| Alkaloids | Tryptophan alkaloids | Carboline alkaloids | 12 |
| Alkaloids | Tryptophan alkaloids | Simple indole alkaloids | 19 |
| Alkaloids | Tryptophan alkaloids | other | 49 |
| Alkaloids | Tyrosine alkaloids | Isoquinoline alkaloids+Tetrahydroisoquinoline alkaloids | 7 |
| Alkaloids | Tyrosine alkaloids | Phenylethylamines | 10 |
| Alkaloids | Tyrosine alkaloids | other | 7 |
| Alkaloids | other | other | 206 |
| Shikimates and phenylpropanoids | Coumarins | Isocoumarins | 7 |
| Shikimates and phenylpropanoids | Coumarins | Simple coumarins | 25 |
| Shikimates and phenylpropanoids | Coumarins | other | 6 |
| Shikimates and phenylpropanoids | Flavonoids | Chalcones | 24 |
| Shikimates and phenylpropanoids | Flavonoids | Flavones | 49 |
| Shikimates and | Flavonoids | Flavonols | 67 |

| | | | |
|---|---|---|---|
| phenylpropanoids | | | |
| Shikimates and phenylpropanoids | Flavonoids | other | 82 |
| Shikimates and phenylpropanoids | Phenolic acids (C6-C1) | Shikimic acids and derivatives+Simple phenolic acids | 13 |
| Shikimates and phenylpropanoids | Phenolic acids (C6-C1) | Simple phenolic acids | 48 |
| Shikimates and phenylpropanoids | Phenolic acids (C6-C1) | other | 15 |
| Shikimates and phenylpropanoids | Phenylpropanoids (C6-C3) | Cinnamic acids and derivatives | 46 |
| Shikimates and phenylpropanoids | Stilbenoids | Monomeric stilbenes | 10 |
| Shikimates and phenylpropanoids | Lignans | Neolignans | 8 |
| Shikimates and phenylpropanoids | other | other | 143 |
| Terpenoids | Monoterpenoids | Secoiridoid monoterpenoids | 9 |
| Terpenoids | Monoterpenoids | other | 29 |
| Terpenoids | Sesquiterpenoids | Eudesmane sesquiterpenoids | 13 |
| Terpenoids | Sesquiterpenoids | Germacrane sesquiterpenoids | 8 |
| Terpenoids | Sesquiterpenoids | other | 61 |
| Terpenoids | Steroids | Bufadienolides | 7 |
| Terpenoids | Steroids | Cardenolides | 3 |
| Terpenoids | Steroids | Estrane steroids | 13 |
| Terpenoids | Steroids | Cholane steroids | 20 |
| Terpenoids | Steroids | Androstane steroids | 21 |
| Terpenoids | Steroids | other | 38 |
| Terpenoids | other | other | 127 |

## Supplementary Figures

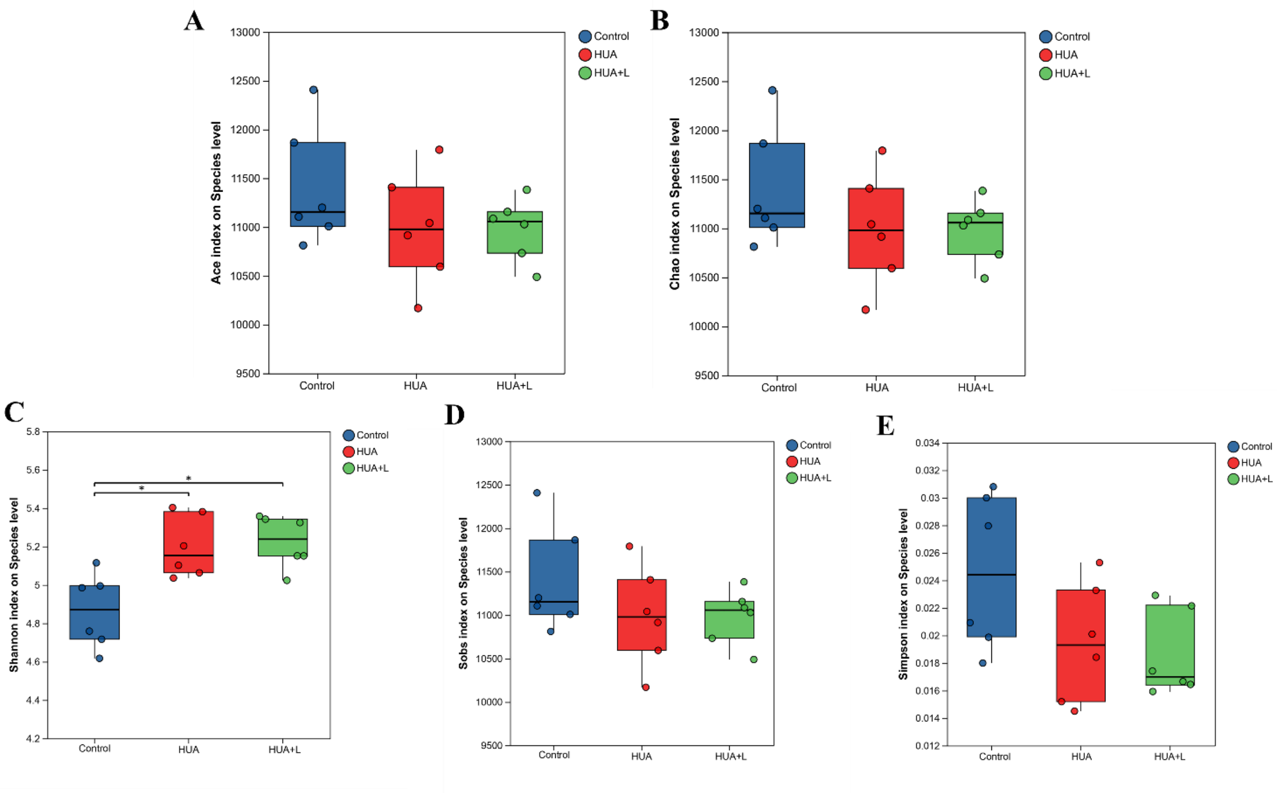


**Supplementary Figure S1.** Alpha diversity box plot analysis. (A) Ace (B) Chao (C) Shamon (D) Sobs (E) Simpson index.
